# Pancreas lineage allocation and specification are regulated by sphingosine-1-phosphate signalling

**DOI:** 10.1371/journal.pbio.2000949

**Published:** 2017-03-01

**Authors:** Ioannis Serafimidis, Eva Rodriguez-Aznar, Mathias Lesche, Kazuaki Yoshioka, Yoh Takuwa, Andreas Dahl, Duojia Pan, Anthony Gavalas

**Affiliations:** 1 Developmental Biology Laboratory, Biomedical Research Foundation of the Academy of Athens, Athens, Greece; 2 Paul Langerhans Institute Dresden of Helmholtz Center Munich at the University Clinic Carl Gustav Carus of TU Dresden, Helmholtz Zentrum München, German Research Center for Environmental Health, Neuherberg, Germany; 3 German Centre for Diabetes Research (DZD), Germany; 4 Deep Sequencing Group SFB655, DFG-Center for Regenerative Therapies Dresden (CRTD), Biotechnology Center (BioZ), Technische Universität Dresden, Dresden, Germany; 5 Department of Physiology, Kanazawa University Graduate School of Medical Sciences, Ishikawa, Japan; 6 Department of Molecular Biology and Genetics, Howard Hughes Medical Institute, Johns Hopkins University School of Medicine, Baltimore, Maryland, United States of America; 7 DFG-Center for Regenerative Therapies Dresden (CRTD), Faculty of Medicine, Technische Universität Dresden, Dresden, Germany; The Francis Crick Institute, UNITED KINGDOM

## Abstract

During development, progenitor expansion, lineage allocation, and implementation of differentiation programs need to be tightly coordinated so that different cell types are generated in the correct numbers for appropriate tissue size and function. Pancreatic dysfunction results in some of the most debilitating and fatal diseases, including pancreatic cancer and diabetes. Several transcription factors regulating pancreas lineage specification have been identified, and Notch signalling has been implicated in lineage allocation, but it remains unclear how these processes are coordinated. Using a combination of genetic approaches, organotypic cultures of embryonic pancreata, and genomics, we found that sphingosine-1-phosphate (S1p), signalling through the G protein coupled receptor (GPCR) S1pr2, plays a key role in pancreas development linking lineage allocation and specification. S1pr2 signalling promotes progenitor survival as well as acinar and endocrine specification. S1pr2-mediated stabilisation of the yes-associated protein (YAP) is essential for endocrine specification, thus linking a regulator of progenitor growth with specification. YAP stabilisation and endocrine cell specification rely on G_αi_ subunits, revealing an unexpected specificity of selected GPCR intracellular signalling components. Finally, we found that S1pr2 signalling posttranscriptionally attenuates Notch signalling levels, thus regulating lineage allocation. Both S1pr2-mediated YAP stabilisation and Notch attenuation are necessary for the specification of the endocrine lineage. These findings identify S1p signalling as a novel key pathway coordinating cell survival, lineage allocation, and specification and linking these processes by regulating YAP levels and Notch signalling. Understanding lineage allocation and specification in the pancreas will shed light in the origins of pancreatic diseases and may suggest novel therapeutic approaches.

## Author summary

The pancreas develops from a field of progenitor cells localised in a restricted region of the embryonic endoderm. These progenitor cells proliferate and eventually differentiate to generate the three distinct lineages comprising the endocrine (which include insulin-producing β cells), acinar, and ductal cells. The molecular pathways implicated in the early generation of pancreas progenitors and their proliferation are well understood. It is also known that the Notch signalling pathway is implicated in sequential binary cell fate decisions that generate the three lineages; however, other signals that may regulate this process remain unknown. Here, we show that a phospholipid, sphingosine-1-phosphate (S1p), generated by the progenitor cells themselves, acts as a signal necessary to define the acinar and endocrine lineage. We observe that in the absence of S1p only duct cells are generated and the survival of pancreas progenitors is compromised. The function of this signalling pathway in the generation of the endocrine cells is two-fold. Firstly, it stabilises yes-associated protein (YAP), a transcriptional gene coactivator known to regulate pancreatic progenitor proliferation, and we show that this stability is necessary for the activation of the endocrine specification program. Secondly, it attenuates Notch signalling, allowing the generation of endocrine and acinar cells. Notch attenuation is necessary for the stabilisation of the transcription factor Ngn3, which is required for the generation of endocrine cells. We conclude that S1p acts as an autocrine signal regulating YAP stabilisation and Notch attenuation to mediate pancreas specification. Understanding lineage allocation and specification in the pancreas will shed light on the origins of pancreatic diseases and may suggest novel therapeutic approaches.

## Introduction

The pancreas is the origin of some of the most debilitating and fatal diseases, including pancreatic cancer and diabetes. Understanding the signalling pathways and gene regulatory networks underlying pancreas development will shed light in the origins of these diseases and suggest novel therapeutic approaches. Mouse reverse genetics and studies in humans have uncovered multiple transcription factors that regulate formation of the pancreatic anlagen and its subsequent expansion, branching morphogenesis, and cell specification into the endocrine, acinar, and ductal lineages [1,2]. Several extracellular signals have also been implicated [2,3], but the molecular mechanisms coordinating lineage allocation with lineage specification remain elusive.

The early pancreatic multipotent progenitor cells (MPCs) emerge at the posterior foregut region of the definitive endoderm and are defined by the expression of the transcription factors Pdx1, Ptf1a, and Sox9 [4–8]. Maintenance of high Notch signalling is necessary for the expansion of MPCs to form a tree-like branched epithelium and prevent early differentiation [9–12]. The Hippo signalling effectors, the transcription factor TEAD1 and its coactivator yes-associated protein (YAP), activate key pancreatic signalling mediators and transcription factors to regulate expansion of pancreatic progenitors [13], but the signal(s) regulating YAP stability are not known. Decreased Notch activity at the tips of the epithelium and the antagonistic functions of Ptf1a and Nkx6 transcription factors delineate the acinar progenitor and endocrine/duct bipotent trunk territories [14–17]. In the trunk, differential Notch signalling enables progenitors to differentiate into ductal and endocrine cells. High Notch levels divert cells to the duct fate through repression of the expression of the Ngn3 transcription factor. Cells escaping high Notch levels induce Ngn3 and become endocrine progenitors [18]. A Notch-mediated posttranslational mechanism for Ngn3 stabilisation has been proposed [19]. Endocrine progenitors provide Notch-dependent and Notch-independent feedback to maintain proliferative growth of the bipotent cell population [20–22]. Despite the central role of Notch signalling, it is not understood whether levels of Notch activity are intrinsically regulated as a function of time or whether unidentified extracellular signals play a role in this process.

We used genetic approaches and organotypic cultures of mouse embryonic pancreata to reveal the implication of sphingosine-1-phosphate (S1p) signalling in progenitor survival, differentiation and lineage allocation. Our data show that S1p signals through the S1p receptor 2 (S1pr2) and YAP to up-regulate connective tissue growth factor (CTGF) that participates in mediating survival of endocrine and acinar progenitors. Signalling specifically through G_αi_ and YAP stabilisation is necessary for endocrine specification. Additionally, S1p signalling attenuates Notch signalling to regulate lineage allocation. Both YAP stabilisation and Notch attenuation are necessary for endocrine and acinar specification. Taken together, the data uncover a novel mode of coordinating lineage allocation and cell specification through S1p signalling and link these processes with tissue growth control.

## Results

### Expression of S1p signalling components peaks during pancreas secondary transition

**Fig 1:**
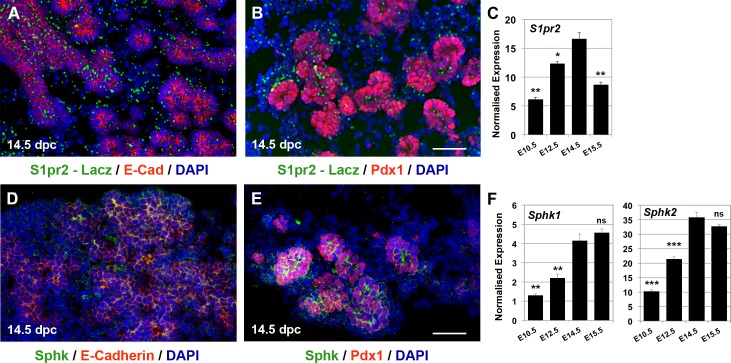
*S1pr2* and *Sphk* expression peaks during pancreas secondary transition. (A-C) β-galactosidase activity by X-gal staining and immunofluorescence on cryosections in heterozygous 14.5 dpc *S1P2* ^*tm1lacz*^ embryos indicated that *S1pr2* is expressed in E-cad^+^ (A) and Pdx1^+^ (B) cells in the developing epithelium and in the mesenchyme (A, B). qPCR analysis showed that *S1pr2* expression peaks at 14.5 dpc and subsequently declines (C). (D-F) Immunofluorescence showed that Sphk localisation is predominantly epithelial, as indicated by the strong Sphk co-staining with E-cadherin (D) and Pdx1 (E). qPCR analysis showed that *Sphk1* and *Sphk2* expression peaks at 14.5 dpc (F). Scale bars, 50 μm; *p < 0.05, **p < 0.01, ***p < 0.001 (C, F) in reference to E14.5 samples; error bars show standard error of the mean (SEM). For raw data please refer to S1 Data.

S1p is a lipid mediator secreted in the extracellular milieu, where it interacts with the S1p G protein-coupled receptors to regulate different cellular responses such as migration, survival, and differentiation [23,24]. Because S1pr2-mediated S1p signalling guides the migration of endocrine progenitors to form islets [25], we decided to examine its implication in earlier stages of pancreas development. We first tracked its expression during mouse pancreas development by following β-galactosidase activity in heterozygous *S1pr2*^*tm1lacz*^ [26] (S3 Table) embryos and by quantitative PCR (qPCR) analysis in wild-type (wt) embryos. Consistent with an early role for this receptor in mesenchymal growth [27], *S1pr2* ^*tm1lacz*^ expression was predominantly mesenchymal from 9.5 days post coitum (dpc) to 12.5 dpc (S1 Fig). During secondary transition, its expression was significantly up-regulated, and in the developing epithelium, it was colocalised with both trunk and tip progenitors (Fig 1A–1C, S1 Fig). By 16.5 dpc, *Lacz* expression was eliminated in nascent endocrine cells and significantly reduced in the rest of the developing organ, whereas it was completely abolished by postnatal day 1 (P1) (S1 Fig). qPCR analysis confirmed this pattern, clearly indicating that *S1pr2* expression in the developing pancreas peaked during secondary transition (Fig 1C). To further investigate the epithelial expression of *S1pr2*, we isolated by fluorescence-activated cell sorting (FACS) the epithelial and mesenchymal components of the developing pancreas at 13.5, 14.5, and 15.5 dpc (S1 Fig). Consistent with the *S1pr2*^*tm1lacz*^ expression pattern, we found that *S1pr2* expression also peaked at 14.5 dpc in the epithelium (S1 Fig).

S1p is a systemically circulating sphingolipid generated by platelets and endothelial cells but it may also be generated in situ. Sphingosine kinases (Sphk) 1 and 2 catalyse the formation of secreted S1p using endogenous cellular sphingosine [28]. Analysis of *Sphk1* and *Sphk2* expression by qPCR showed that their expression peaked during secondary transition and suggested a predominantly epithelial expression (Fig 1F, S1 Fig). Immunostainings at 12.5, 14.5. and 16.5 dpc confirmed the predominantly epithelial expression of Sphk during that time (Fig 1D and 1E and S1 Fig).

Taken together, the temporal expression profile of *S1pr2* and *Sphks* suggested that this signalling pathway may act as an autocrine signal to mediate pancreas specification. Expression of both *S1pr2* and *Sphks* in the developing epithelium peaked at 14.5 dpc, suggesting that this time point was optimal to address their possible role in pancreas specification.

### Developmental delay of the *S1pr2* null pancreata reveals key role in lineage specification

To address the role of *S1pr2* in pancreas development, we first compared the RNA Seq gene expression profiles of *S1pr2*^*tm1Rlp*^ (S3 Table) null [29] embryonic pancreata (S2 Fig) with those of 13.5, 14.5, and 15.5 dpc wt embryonic pancreata.

**Fig 2:**
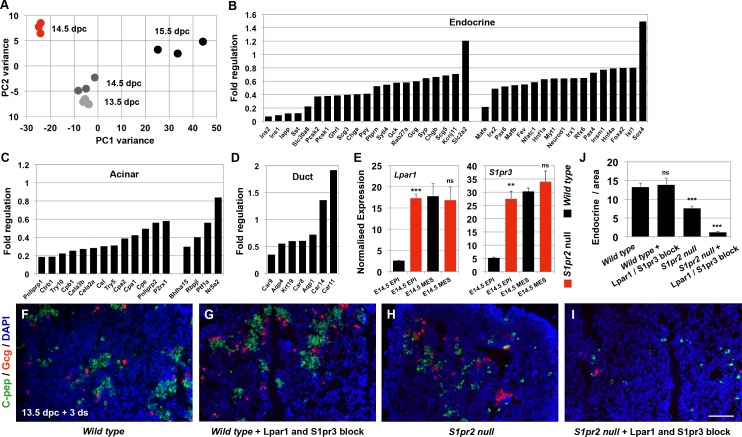
*S1pr2* null pancreata are developmentally delayed but up-regulate *Lpar1* and *S1pr3*. (A-D) PCA of RNA Seq gene expression profiles of 14.5 dpc *S1pr2*^*tm1Rlp*^ null pancreata (in red) and 13.5, 14.5, and 15.5 dpc wt pancreata (in shades of black). PC1 shows developmental time because it shows the highest variability among wt samples and there is only a small variance in PC2. *S1pr2* null pancreata cluster away from their wt counterparts, suggesting that they were developmentally delayed (A). Analysis of these RNA Seq data revealed that endocrine- (B) and acinar- (C) specific genes were strongly repressed in *S1pr2* null pancreata at 14.5 dpc as compared to 14.5 dpc wt controls, whereas expression of duct-specific genes was less affected (D). (E) qPCR analysis of *Lpar1* and *S1pr3* expression in the FACS-isolated epithelial and mesenchymal components of 14.5 dpc wt (black bars) and *S1pr2* null (red bars) pancreata indicated that expression of both *Lpar1* and *S1pr3* was selectively up-regulated in the *S1pr2* null epithelium by 7- and 5-fold, respectively. (F-J) air–liquid interface (ALI) cultures of 13.5 dpc wt embryonic pancreata for 3 d results in the generation of several C-pep^+^ and Gcg^+^ cells (F), and combined treatment with 20 μM and 50 μM of the specific Lpar1 and S1pr3 inhibitors, respectively, had no effect (G, J). In contrast, the already reduced number of endocrine cells in the *S1pr2* nulls (H, J) is further reduced upon addition of the two antagonists in these concentrations (I, J). *Padj* for all genes <0.05 except Foxa2 where *Padj* <0.1 (B-D); **p < 0.01, ***p < 0.001, ns not significant in reference to corresponding epithelial or mesenchymal values (E, F); error bars in E show SEM; error bars in J show standard deviation (SD); scale bars are at 80 μm. For raw data, please refer to the S1 Data file.

Principal component analysis (PCA) suggested that *S1pr2* null pancreata were developmentally delayed (Fig 2A). Endocrine and acinar lineage commitment genes were strongly down-regulated. Ductal specification was much less affected, and the expression of important duct markers such as *Hnf1b*, *Car2*, and *Slc9a1* was not affected (Fig 2B–2D, S1 Table). Surprisingly, expression of transcription factors and other genes implicated in epithelial progenitor specification and maintenance was not significantly affected (S2 Fig, S1 Table) [30]. Consistent with that, organ size, epithelial proliferation assessed by pH3 immunofluorescence, and cell survival assessed by TUNEL assays were not affected (S2 Fig). These findings suggested that expansion of the progenitor population in the *S1pr2*^*tm1Rlp*^ null pancreata was not affected, but lineage commitment was severely delayed.

*S1pr2* null embryos develop to full term but show perinatal mortality and display a range of phenotypes, particularly in the hematopoietic and vascular systems [26,29]. Surprisingly, immunofluorescence analysis of *S1pr2* ^*tm1Rlp*^ null pancreata at P1 showed no major defects in the development of the endocrine, acinar, and ductal lineages. Additionally, fasting glucose levels and glucose tolerance tests in the surviving adults failed to uncover glucose homeostasis defects, suggesting no gross alterations, at least concerning endocrine cell function (S2 Fig).

To investigate whether systemic signals were necessary for functional compensation, we used serum free air–liquid interface (ALI) cultures of embryonic pancreata. ALI cultures mimic normal embryonic development and thus also give a means to temporally manipulate signalling conditions and elucidate their effects on lineage specification [25,31]. Wt 14.5 dpc pancreata in ALI cultures continue their developmental program over the course of 6 d, giving rise to acinar, endocrine, and duct cells (S3 Fig). However, *S1pr2*^*tm1Rlp*^ 14.5 dpc null pancreata failed to develop normally, because both the endocrine and acinar lineages were severely compromised and there was a strong increase in the number of duct-like cells (S3 Fig). This was consistent with the strong down-regulation of endocrine and acinar differentiation markers at 14.5 dpc (Fig 2B and 2C) and in contrast to the eventually normal development of *S1pr2*^*tm1Rlp*^ null pancreata in utero. We next looked for up-regulated related receptors that may mediate functional compensation of the *S1pr2* null mutation and rescue the early pancreatic phenotype of the *S1pr2* nulls.

*S1pr2* belongs to the family of the closely related lysophospholipid (LPL) receptors (LPLrs) that have similar expression patterns and overlapping functions [32,33]. RNA Seq data showed that only *S1pr3* and *lysophosphatidic receptor 1 (Lpar1)*, which are functionally related to *S1pr2* [32], were expressed to any appreciable extent and to levels comparable to that of *S1pr2* in the 14.5 wt pancreata (S1 Table). Interestingly, *S1pr3* and *Lpar1* expression increased in the 14.5 dpc *S1pr2*^*tm1Rlp*^ null total pancreata (S2 Fig). To independently confirm that this up-regulation was a consequence of abrogating S1pr2 signalling, we acutely blocked S1pr2 function by injecting intraperitoneally (ip) pregnant dams at 13.5, 14.5, and 15.5 dpc with either 2 or 5 mg/Kg body weight of the specific S1pr2 antagonist JTE013 [34]. In both treatments, expression of both *S1pr3* and *Lpar1* was up-regulated in 16.5 dpc pancreata (S2 Fig). Expression of *S1pr3* and *Lpar1* is primarily mesenchymal in 13.5, 14.5, and 15.5 dpc wt pancreata (S2 Fig), but, strikingly, their expression was selectively up-regulated, by 5- and 7-fold, respectively, in the epithelium of 14.5 dpc *S1pr2* null pancreata (Fig 2E and S2 Fig).

To establish that these receptors mediate the recovery of the endocrine lineage, we blocked their function in ALI cultures by applying for 3 d the specific S1pr3 and Lpar1 inhibitors VPC23019 and Ki16425 at 50 μM and 20 μM, respectively, in ALI cultures of 13.5 dpc wt and *S1pr2* null pancreata. Either inhibitor on its own was not sufficient to block endocrine specification, but when used in combination, they essentially shut off endocrine specification, specifically in S1pr2 null pancreata but not in wt pancreata (Fig 2F–2J), demonstrating that *S1pr3* and *Lpar1* mediate the functional compensation in the *S1pr2* nulls.

These findings established an important role for S1p signalling in the specification of pancreas progenitors and uncovered extensive functional compensation among specific LPL receptors in pancreas development.

### S1pr2 signalling has a dual role in promoting survival and commitment of pancreas progenitors

To fully address the role of S1pr2 signalling in pancreas development, we decided to isolate systemic signals mediating the functional compensation in *S1pr2* null embryos using ALI cultures. The presence of 15 μM JTE013 in ALI cultures of 14.5 dpc *S1pr2* null pancreata did not alter their growth, survival, and differentiation patterns, further confirming its specificity (S3 Fig). In contrast, blocking S1pr2 function in wt pancreata resulted in obvious morphological defects, most notably the absence of light-reflective, dense cell clusters (S3 Fig).

**Fig 3:**
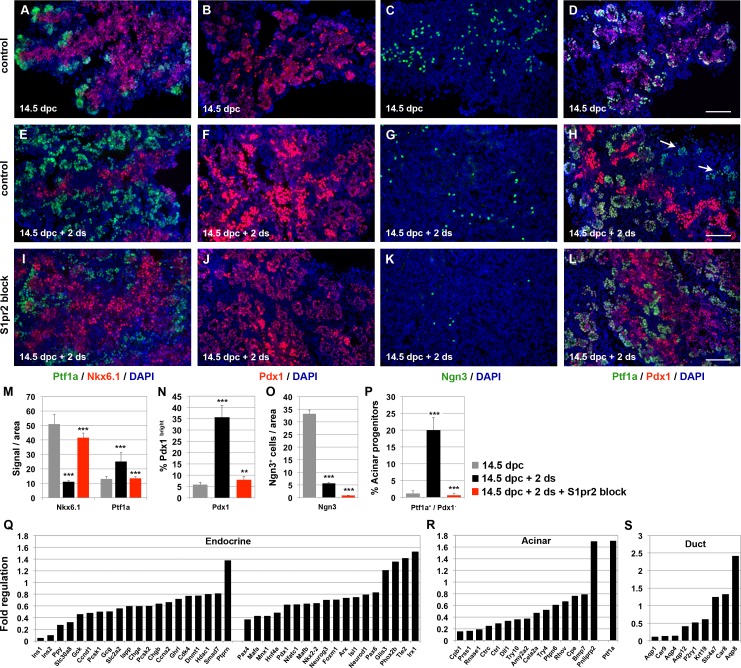
S1pr2 signalling promotes commitment of pancreas progenitors. (A-H) Immunofluorescence analysis of embryonic pancreata at 14.5 dpc and after 2 d in ALI cultures (14.5 dpc + 2 d) shows the progression of the developmental program. This was apparent by the reduction of Nkx6-1^+^ (A, E) and Ngn3^+^ (C, G) progenitor cells, the corresponding increase of strongly labelled Pdx1^+^ cells (B, F) and the appearance of Ptf1a^+^/Pdx^-^ cells (D, H; arrows in H). (I-L) Immunofluorescence analysis at 14.5 dpc + 2 d in the absence of S1pr2 signalling clearly showed that the Nkx6-1^+^ population was expanded (I), Ngn3^+^ cells (K) and strongly labelled Pdx1^+^ cells (J) were dramatically reduced, and Ptf1a^+^/Pdx1^-^ cells were lost (L). (M-P) Quantitation of Nkx6.1^+^and Ptf1a^+^ cells (M), Pdx1^+^ cells (N), Ngn3^+^ cells (O), and acinar progenitors (Ptf1a^+^ / Pdx1^-^) (P) in 14.5 dpc pancreata, 14.5 dpc pancreata cultured in ALI for 2 d in the absence or presence of 15 uM of the S1pr2 specific inhibitor JTE013. Statistical significance is shown for the comparisons between 14.5 dpc and 14.5 dpc + 2 d stainings (14.5 dpc + 2 d bar) as well as between 14.5 dpc + 2 d with and without S1pr2 block (14.5 dpc + 2 d + S1pr2 block bar). (Q-S) RNA Seq gene expression analysis on 14.5 dpc pancreatic explants after 2 d in ALI culture with S1pr2 signalling blocked revealed a coordinated repression of transcription factors and terminal differentiation markers of the endocrine (Q) and acinar (R) lineages and, to a lesser extent, of the ductal lineage (S) compared to untreated control explants at 14.5 dpc + 2 d. Scale bars, 80 μm; **p < 0.01, ***p < 0.001, *Padj* for all genes <0.05; error bars M, N, P show SD; error bars in O show SEM. For raw data, please refer to the S1 Data file.

The progression of the developmental program in ALI cultures of wt 14.5 dpc pancreata is apparent 2 d following culture initiation. The segregation of the endocrine and ductal lineages from the Nkx6.1^+^ bipotent progenitors has progressed and is shown by the reduction in the numbers of Nkx6.1^+^ and Ngn3^+^ cells, the increase of strongly labelled Pdx1^+^ cells, and the appearance of numerous C-pep^+^ and Gcg^+^ as well as CK19^+^ cells. Acinar cells become numerous, as seen by the appearance of Ptf1a^+^/Pdx1^-^ and Amy^+^ cells (compare Fig 3A–3D with 3E–3H, see also S4 Fig and Fig 3M–3P) [16]. However, in the absence of S1pr2 signalling, development of both acinar and endocrine lineages was blocked, as shown by the loss of Amy^+^ and C-Pep^+^, whereas the development of CK19^+^ duct-like cells did not appear affected (S4 Fig). These findings correlated with the persistence of a large number of Nkx6.1^+^ cells, the loss of Ptf1a^+^/Pdx1^-^ cells, the reduction of strongly labelled Pdx1^+^ cells as well as the dramatic reduction in the number of Ngn3^+^ cells (compare Fig 3E–3H with 3I–3L, see also S4 Fig and Fig 3M–3P). Additionally, there was a marked decrease of epithelial proliferation and the appearance of apoptotic cells (S4 Fig).

To reveal the full extent of the changes, we analysed the transcriptome of ALI pancreatic explants cultured for 2 d. Similarly to the transcriptome analysis of *S1pr2* null pancreata at 14.5 dpc, the expression of progenitor markers, upon S1pr2 blocking with 15 μM of JTE013, was only mildly affected, with the striking exception of the transcription factor *Nkx6*.*2* and the Notch membrane-bound ligand *Dll1*, two genes that are important for the segregation of the bipotent trunk population from the acinar progenitor pool and Notch-mediated lineage segregation (S4 Fig, S1 Table) [16,18]. On the other hand, and in agreement with the transcriptome analysis of *S1pr2* null pancreata at 14.5 dpc, there was coordinated repression of transcription factors and terminal differentiation markers of the endocrine and acinar lineages. In contrast, the ductal lineage was much less affected because, similarly to *S1pr2* null phenotype at 14.5, expression of several important genes, such as *Hnf1b*, *Car2*, and *Slc9a1*, was not affected (Fig 3Q–3S, S1 Table). Consistent with an important role of S1pr2 signalling for lineage commitment during secondary transition, the total number of regulated genes in its absence increased by 2.5-fold at 14.5 dpc + 2 d (S4 Fig).

**Fig 4:**
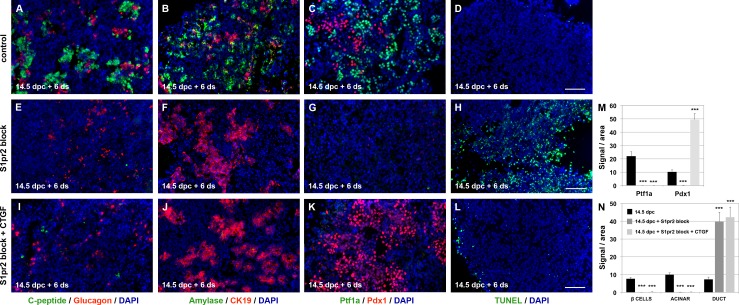
S1pr2 signalling promotes survival and lineage specification of pancreas progenitors. (A-H, M, N) Immunofluorescence analysis showed that S1pr2 block with 15 μM JTE013 in ALI cultures of 14.5 dpc embryonic pancreata for 6 d (14.5 dpc + 6 d) lead to the elimination of C-peptide^+^ endocrine cells (A, E, N) and Amylase^+^ acinar cells (B, F, N) and to a greatly expanded number of CK19^+^ duct-like cells (B, F, N). Markers of mature β cells (Pdx1) and acinar cells (Ptf1a) were also eliminated upon S1pr2 block (C, G, M), and cell survival was severely compromised, as shown by TUNEL immunofluorescence analysis (D, H). (I-L, M, N) Immunofluorescence and TUNEL analysis showed that addition of CTGF in the ALI cultures along with the S1pr2 block virtually eliminated cell death (L), without reversing the loss of C-peptide^+^ endocrine (I, N), Amylase^+^ acinar cells (J, N). A large number of CK19^+^ duct-like cells (J, N) and Pdx1^+^ cells (K, M) persisted. Scale bars, 80 μm (A, B, D-F, H-J, L) and 70 μm (C, G, K); ***p < 0.001; error bars show SD. For raw data please refer to the S1 Data file.

After 6 d in culture in the absence of S1pr2 signalling, beta cells and acinar cells were virtually eliminated, and the explants were taken over by a greatly expanded number of CK19^+^ duct-like cells (compare Fig 4A–4C, Fig 4E–4G and 4N). Additionally, cell survival was severely compromised at that stage, as shown by TUNEL assays (Fig 4D and 4H). To establish that S1p is the ligand mediating these effects, we supplemented the S1pr2-blocked ALI cultures with 20 μM S1p and achieved a rescue of both the acinar and endocrine lineages (S3 Fig, S7 Fig).

These findings showed that S1p signalling, through S1pr2, was necessary for both cell survival and lineage commitment. To address whether these effects were distinct from one another, we sought means to rescue the cell survival independently of cell specification. CTGF was a plausible candidate to participate in mediating survival, as it has been implicated as such a signal downstream of S1p signalling and it is expressed in the developing pancreas in both the epithelium and the mesenchyme (S5 Fig) [35]. Importantly, its expression was significantly repressed (3.5-fold) upon blocking S1pr2 signalling for 2 d in ALI 14.5 dpc explants (S5 Fig). Addition of 50 ng/ml CTGF at the onset of the ALI culture in the absence of S1pr2 signalling eliminated cell death 6 d later (Fig 4D, 4H and 4L). However, this was not sufficient to reverse the early differentiation block, and results were very similar in this respect. After 2 d in culture, there were again very few Ngn3^+^ cells, no Ptf1a^+^/Pdx1^-^ cells, the number of strongly labelled Pdx1^+^ cells did not increase compared to the 14.5 dpc stage, and proliferation rates in the epithelium remained low (compare Fig 3G, 3K, 3H and 3L with S5 Fig and S5 Fig with S4 Fig). Consistent with that, after 6 d in culture, the endocrine cells, beta cells in particular, and acinar cells were again missing, whereas the numbers of duct-like cells remained greatly expanded (compare Fig 4A and 4B to 4E, 4F, 4I and 4J, see also Fig 4N). These findings, the loss of Ptf1a^+^ cells, and the persistence of a large number of Pdx1^+^ and Nkx6.1^+^ cells (Fig 4C, 4G and 4K, S5 Fig and Fig 4M) suggest that cells are stabilised in a trunk bipotential identity when S1pr2 signalling is blocked but survival is rescued by CTGF. To further corroborate that cell survival was mediated independently of cell fate commitment, we compared gene expression changes of 14.5 + 2 d ALI embryonic pancreata cultured in conditions of S1pr2 block in the presence or absence of 50 ng/ml CTGF. Gene expression changes of progenitor, endocrine, acinar, and duct markers [30] were strikingly similar in the two conditions as compared to untreated controls (S5 Fig, S1 Table). Furthermore, PCA showed that S1pr2 blocked explants with or without CTGF clustered much closer together (PC2, 16% variance) than with control pancreata (PC1, 68% variance) (S5 Fig). To establish that S1p signalling regulates expression of *CTGF*, we used 20 μM of S1p in combination with 15 μM of JTE013 in 14.5 dpc ALI cultures and showed that *CTGF* expression was restored to normal levels by S1p (S5O Fig).

These data showed that S1p signalling through S1pr2 mediates cell fate commitment to the endocrine and acinar lineages. Independently of that, it promotes progenitor cell survival at least partly through up-regulation of CTGF.

### A novel role for G_αi_ subunits in mediating endocrine pancreas specification

**Fig 5:**
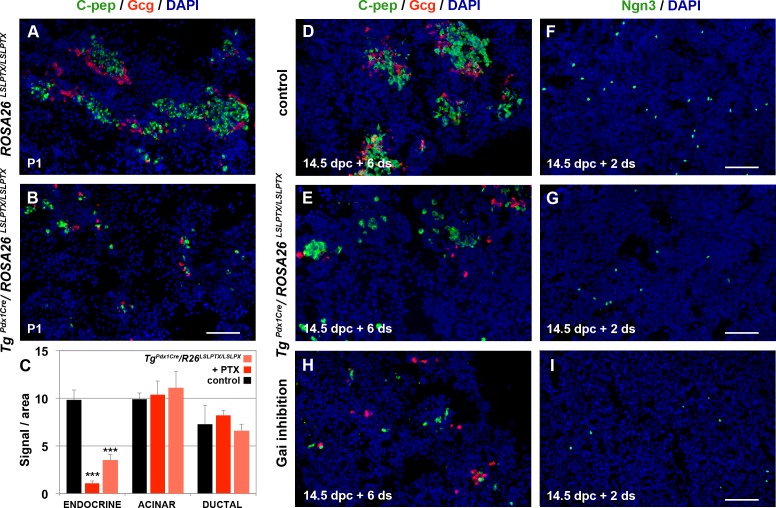
G_αi_ subunits mediate endocrine pancreas specification. (A-I) Activation of PTX expression from two alleles of the *ROSA26* ^*LSLPTX*^ transgene using the *Tg*^*Pdx1Cre*^ driver resulted in a striking loss of C-pep^+^ and Gcg^+^ cells at P1, as shown by immunofluorescence analysis (A, B; Quantitations are provided in S6L Fig). ALI cultures of 14.5 dpc embryonic pancreata expressing PTX from two alleles in Pdx1^+^ progenitors also resulted in reduced numbers of C-pep^+^ and Gcg^+^ cells after 6 d in culture (C, D, E), preceded by an equivalent reduction in the number of Ngn3^+^ cells at 2 d (F, G). ALI cultures of 14.5 dpc wt pancreata in the presence of 10 μg/ml of PTX showed a dramatic reduction of Ngn3^+^ cells (I) and the total number of C-pep^+^ and Gcg^+^ cells (C, H) at 2 and 6 d, respectively. Neither acinar (Amy^+^ cells) nor ductal (CK19^+^ cells) specification was affected by PTX in either the genetic or the pharmacological approach (C). Scale bars, 80 μm (A, B) and 70 μm (D-I); ***p < 0.001 in reference to control ALI cultures (C); error bars show SD. For raw data please refer to the S1 Data file.

As shown above, genetic inactivation of *S1pr2* results in strong developmental delay, particularly for the endocrine and acinar lineage specification, but this is compensated for by up-regulation of the expression of the related receptors *S1pr3* and *Lpar1* specifically in the epithelium. To bypass this compensation and identify the downstream components of S1pr2 signalling, we first targeted the function of G_αi_ subunits that are common mediators among these receptors [32] and can be specifically inactivated by pertussis toxin A (PTX)-mediated adenosine diphosphate (ADP)-ribosylation [36,37]. We previously showed that conditional expression of a single allele of the *ROSA26*^*LSLPTX*^ transgene [38] (S3 Table) in endocrine progenitors using the *Ngn3-Cre* driver mouse line *Tg*^*Ngn3Cre*^ [39] (S3 Table) or a low (5 μg/ml) PTX dose in embryonic pancreatic explants resulted in disruption of endocrine cell migration to form islets [25]. Expression of a single allele of the *ROSA26*^*LSLPTX*^ transgene in epithelial progenitors using the *Pdx1-Cre* driver *Tg*^*Pdx1Cre*^ [40] (S3 Table) had a similar effect (S6 Fig), but neither treatment had any effect on pancreas lineage specification [25]. Strikingly, activation of two alleles of the *ROSA26*^*LSLPTX*^ transgene using the same driver resulted in specific and strong loss of C-pep^+^ and Gcg^+^ endocrine but not Amy^+^ acinar or CK19^+^ duct cells at P1 (Fig 5A and 5B, S6 Fig). ALI cultures of 14.5 dpc embryonic pancreata expressing two PTX alleles in Pdx1^+^ progenitors also generated very few endocrine cells after 6 d in culture without loss of Amy^+^ acinar or CK19^+^ duct cells (Fig 5C–5E, S6 Fig). Loss of endocrine cells was preceded by a dramatic reduction in the number of Ngn3^+^ cells (Fig 5F and 5G, S6 Fig).

To assess whether loss of the Ngn3^+^ progenitors in the *Tg*^*Pdx1Cre*^*/ROSA26*^*LSLPTX/LSLPTX*^ embryos was due to disruption of signalling events directly responsible for *Ngn3* induction or defective competence of Pdx1^+^ progenitors acquired in earlier stages of pancreas development, we cultured 14.5 dpc wt embryonic pancreata in ALI culture in the presence of 10 μg/ml dose of PTX. The effects of this treatment closely matched the genetic experiment, as the number of C-Pep^+^ and Gcg^+^ cells was also dramatically reduced, but the number of Amy^+^ acinar or CK19^+^ duct cells was not affected (Fig 5C and 5H, S6 Fig). Again, this was preceded by a dramatic reduction in the number of Ngn3^+^ cells (Fig 5I, S6 Fig). Thus, G_αi_-mediated signalling controls both endocrine specification and migration in a dose-dependent manner. Irrespective of the dose, neither acinar nor ductal specification was affected in either the genetic or ALI paradigms.

The data described so far showed that S1p signalling through S1pr2 plays an important role in pancreas progenitor survival at least partly through *CTGF* transcriptional activation and in triggering progenitor specification. Additionally, our findings show that endocrine specification in particular is specifically mediated by G_αi_ subunits.

### YAP mediates S1pr2 signalling and is important for endocrine specification

We next sought to determine the transcriptional mediator of this signalling cascade. The transcriptional responses following S1p activation are not fully understood, but recent work has shown that S1p receptors can stabilise the transcriptional coactivator YAP, a central player in tissue growth control [41–45]. YAP is a component of the Hippo signalling cascade that phosphorylates it, leading to its retention in the cytoplasm and its degradation by the proteasome [46]. Additionally, CTGF is a direct target of YAP transcriptional activation, mediating cell survival in different cellular contexts [47–49]. Importantly, the TEAD obligatory YAP transcriptional partners have been shown to bind to multiple enhancer elements of genes implicated in pancreas development [13].

**Fig 6:**
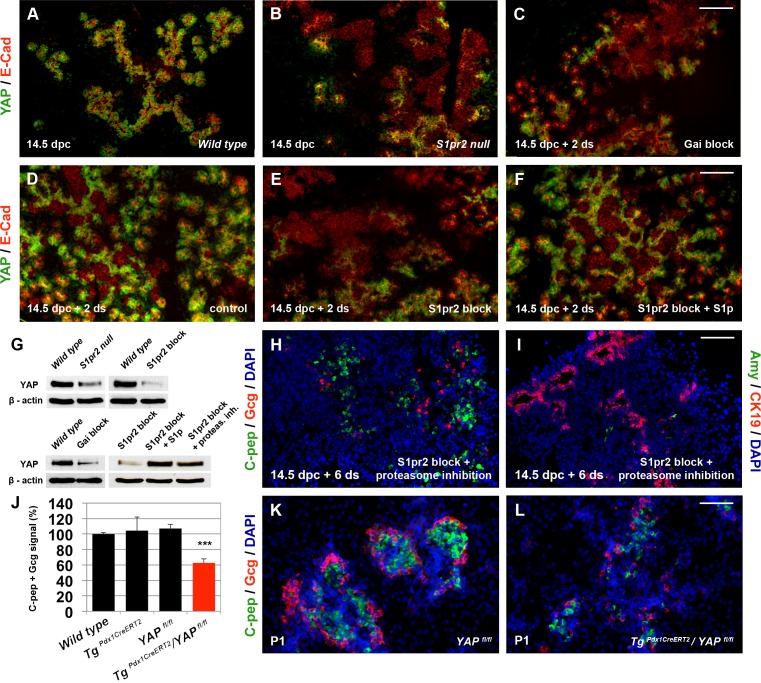
YAP mediates G_αi_-dependent S1pr2 signalling and is essential for endocrine specification. (A-B) Immunofluorescence analysis of embryonic pancreata at 14.5 dpc showed that YAP protein is specifically expressed in E-cadherin^+^ epithelial cells (A) and that it is dramatically reduced in S1pr2 nulls (B). (C-F) YAP expression is retained in the epithelium of 14.5 ALI embryonic pancreas explants cultured for 2 d (D) but dramatically reduced upon either S1pr2 block by 15 μM JTE013 (E) or G_αi_ inactivation by 10 μg/ml PTX (C). YAP expression levels are restored in JTE013-treated pancreata by 20 μM of S1p (F). (G) YAP protein levels detected by western blot were significantly reduced in *S1pr2* null pancreata at 14.5 dpc and in 14.5 dpc ALI cultures subjected to S1pr2 blocking with 15 μM JTE013 for 2 d or subjected to G_αi_ inhibition by 10 μg/ml of PTX. YAP protein levels were restored in JTE013 treated pancreata by 20 μM of S1p or 1 uM of the proteasome inhibitor MG132. (H, I) Immunofluorescence analysis showed that C-pep^+^ and Gcg^+^ cells were restored in JTE013-treated 14.5 dpc + 6 d ALI cultures in the presence of 1 μM MG132 for the first 2 d (H). Amy^+^ cells, however, were not restored under these conditions (I) and CK19 expression remained high (I). Quantitations are provided in S7A Fig. (J–L) Inactivation of YAP in pancreas progenitors using the *YAP*^*fl/fl*^ conditional allele, the *Tg*^*Pdx1CreERT2*^ driver, ip tamoxifen injections, and immunofluorescence analysis showed a nearly 40% reduction in the number of endocrine C-pep^+^ and Gcg^+^ cells compared to controls at P1. Scale bars, 80 μm (B-F, H, I) and 70 μm (K, L); ***p < 0.001, ns, not significant in reference to E14.5 samples (A) and wt embryonic pancreata; error bars show SEM with the exception of J, for which they show SD. For raw data, please refer to the S1 Data file.

Expression of *YAP* can be detected from the early stages of pancreas development, but, similarly to *S1pr2* and *Sphk* expression, it peaks during secondary transition, and transcript levels are significantly higher in the epithelium (S7 Fig). Immunostaining with E-cadherin clearly showed that the protein is stabilised and detectable only in the epithelium (Fig 6A). Therefore, we asked whether S1pr2 signalling regulates YAP levels and, therefore, YAP-mediated transcriptional regulation. RNA Seq and qPCR results clearly showed that *YAP* expression was not affected in either *S1pr2* nulls, S1pr2 blocked ALI cultures, or G_αi_ blocked ALI cultures (S7 Fig). In contrast, YAP protein levels were significantly reduced in both *S1pr2* null pancreata at 14.5 dpc and 14.5 pancreatic explants subjected to S1pr2 signalling block for 2 d in culture (Fig 6A, 6B, 6D, 6E and 6G). YAP target genes such as *CTGF*, *Cdk6*, and *Ccnd1* identified in other contexts [47,50,51] as well as *Sox9* and *CTGF* in pancreas development [13] were transcriptionally down-regulated in S1pr2 blocked explants (S2 Table). To show direct dependence of YAP stability upon S1p signalling, we titrated the amount of exogenous S1p and found that 20 μM S1p in explants treated with 15 μM JTE013 was sufficient to rescue YAP protein stability (Fig 6D–6F and 6G). The determined binding affinities are slightly higher for S1p ^34, 52^ and therefore in these concentrations S1p is in small molecular excess. Additionally, S1p, being the natural ligand, should diffuse faster in the explant tissue. This finding further confirmed that YAP protein stability depends on S1p signalling through S1pr2.

S1pr2 signalling can be conveyed through G_αq_, G_α_12/13, and G_αi_ subunits [52], and we sought to determine whether selective blocking of each of these Ga subunits would affect YAP protein stability. Blocking of the G_αq_ subunits with 50 uM of the specific G_αq_ antagonist-2A (GP2A) [53] or blocking the function of G_α_12/13 subunits with 5 ug/ml of the C3 exoenzyme [54] in explants had no effect on YAP stability (S7 Fig). In contrast, inactivation of G_αi_ subunits by 10 μg/ml PTX resulted in a dramatic loss of YAP protein stabilisation, showing that signalling specifically through G_αi_ subunits is an important component of YAP stabilisation (Fig 6C and 6G).

To address the potential role of YAP in pancreas specification, we first sought to rescue the protein stability in ALI embryonic pancreas explants where S1pr2 signalling was blocked. Addition of 2 d of 1 μM of the reversible proteasome inhibitor MG132, used previously to restore YAP stability [55], resulted in the YAP stabilisation (Fig 6G). Strikingly, this restored specifically endocrine but not acinar specification (Fig 6H and 6I, S7 Fig), suggesting that S1pr2 signalling acts through G_αi_ and YAP stabilisation to promote the endocrine lineage. To directly address this, we used the *Tg*^*Pdx1CreERT2*^ Cre driver and the *YAP*^*fl/fl*^ allele (S3 Table) to temporally inactivate YAP and bypass its early role in the expansion of the pancreatic epithelium [13]. Following tamoxifen administration permissible for normal pregnancy and embryo development [56], the *Tg*^*Pdx1CreERT2*^ [57] allele partially labelled all three lineages in the presence of a conditional *ROSA26*^*LSLtdTomato*^ reporter allele (S3 Table) at similar efficiencies (S7 Fig). Thus we used *Tg*^*Pdx1CreERT2*^ and tamoxifen administration to inactivate YAP (S7 Fig) in a temporally delayed manner. Whereas there was no effect on the number of Ngn3^+^ cells at 14.5 dpc (S7 Fig), the number of endocrine Cpeptide^+^ and Gcg^+^ cells was reduced by nearly 40% at P1 compared to controls (Fig 6J–6L). The number of Amy^+^ acinar and CK19^+^ duct cells was not affected, demonstrating the specific role of YAP in endocrine specification (S7 Fig). There was no cell death in these pancreata, suggesting that the remaining YAP^+^ epithelial cells generated sufficient secreted CTGF and/or other secreted factors to maintain cell survival. To further confirm that S1p signalling acts through YAP in endocrine specification, we treated 14.5 dpc YAP-deleted pancreata with 20 μM S1p for 6 d in culture. This treatment failed to restore the loss of endocrine cells, confirming that YAP mediates the S1p effects on endocrine specification (S7 Fig).

Taken together, these results reveal a distinct mechanism whereby S1p signalling acts through G_αi_ subunits to stabilise YAP, which in turn is necessary for endocrine specification. Importantly, YAP destabilisation does not affect the number of Ngn3^+^ cells, suggesting that a distinct S1p-dependent pathway regulates Ngn3 expression.

### S1pr2-mediated attenuation of Notch signalling is necessary for endocrine and acinar differentiation

**Fig 7:**
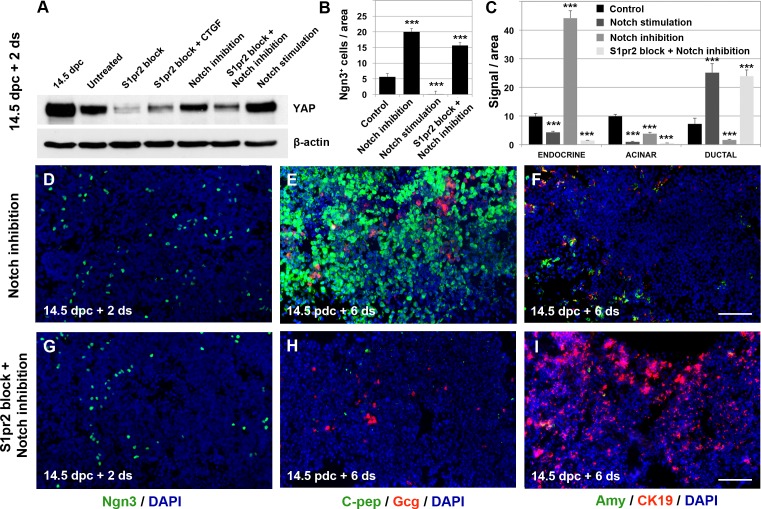
S1pr2-mediated Notch attenuation triggers endocrine and acinar differentiation. (A-C) Stimulation of Notch signalling with 10 μM DSL in 14.5 dpc + 2 d ALI cultures did not affect YAP protein levels, as shown by western blot analysis (A) but resulted in elimination of Ngn3^+^ cells (B, immunostaining in S8A Fig) and a significant reduction in the number of endocrine and acinar cells in favour of an expanded population of ductal cells (C, immunostainings in S8B and S8C Fig). (A-C, D-F) Inhibition of Notch signalling with 10 μM DAPT in 14.5 dpc + 2 d ALI cultures had no effect on YAP protein levels (A) but resulted in a large increase in the number of Ngn3^+^ (B, D), C-pep^+^, and Gcg^+^ (E, C) cells at the expense of Amy^+^ and CK19^+^ cells (F, C), as shown by immunofluorescence. (A-C, G-I). Simultaneous inhibition of Notch and S1pr2 signalling in 14.5 dpc + 2 d ALI cultures transiently resulted in a large increase in the number of Ngn3^+^ cells (B, G) shown by immunofluorescence. However, immunofluorescence analysis showed that C-pep^+^, Gcg^+^ (H, C), and Amy^+^ (I, C) cells were eventually eliminated in favour of an expanded population of CK19^+^ cells (I, C). Absence of terminally differentiated endocrine and acinar cells can be attributed to a significant reduction in YAP protein levels observed under these conditions (A). Scale bars, 80 μm (D-I); ***p < 0.001 in reference to control ALI cultures (B); error bars in B show SEM; error bars in C show SD. For raw data, please refer to the S1 Data file.

Disruption of S1pr2 signalling dramatically reduced the number of Ngn3^+^ cells (Fig 3G, 3K and 3O). YAP destabilisation could not account for this effect, as shown above by the conditional inactivation of *YAP*. Additionally, after S1pr2 signalling disruption, *Ngn3* gene expression was only marginally affected, and there was a large increase in the number of duct-like cells (Figs 3G, 3K, 3O, 3Q, 4B, 4F and 4N). Both of these effects could be a result of high Notch activity that has been shown to destabilise Ngn3 and induce the formation of supernumerary duct cells [18,19,58,59]. To investigate this, we activated Notch signalling above the endogenous levels using 10 μM of the soluble Notch ligand peptide Delta-Serrate/lag-2 (DSL) in ALI cultures of 14.5 dpc pancreata. This peptide is derived from the Jag-1 ligand domain, and it also corresponds to the conserved, receptor binding part of the other Notch ligands, thus eliciting canonical Notch signalling [60,61]. This activation resulted in a very similar phenotype to that of blocking S1pr2 signalling, disrupting endocrine and acinar specification. Ngn3^+^ cells were essentially eliminated (S8 Fig and Fig 7B), and the number of endocrine and acinar cells was drastically reduced in favour of a greatly expanded population of duct-like cells (compare Fig 4A, 4B, 4E and 4F with S8 Fig, see also Fig 7C).

**Fig 8:**
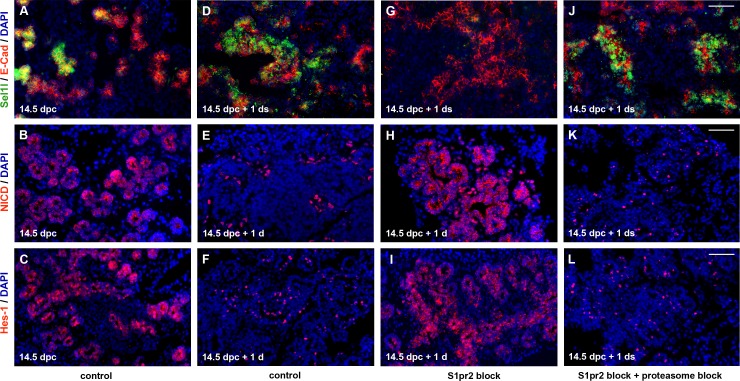
S1pr2 signalling attenuates Notch signalling. (A-F) Immunofluorescence analysis showed that Sel1l is strongly expressed in 14.5 dpc pancreata maintained for 1 d (14.5 dpc + 1 d) in ALI cultures compared to 14.5 dpc pancreata (A, D), and this correlated with a drop in the expression levels of NICD (B, E) and Hes1 (C, F). (G-I) S1pr2 signalling block with 15 μM JTE013 in 14.5 dpc + 1 d ALI cultures resulted in loss of Sel1l expression (G), concomitant with maintenance of high NICD (H) and Hes1 (I) expression. (J-L) Sel1l expression was restored in the absence of S1pr2 signalling by 1 μM of the specific proteasome inhibitor MG132 in 14.5 dpc + 1 d ALI cultures (J), and this was sufficient to restore attenuation of both NICD (K) and Hes1 (L) expression. Scale bars, 50μm (A-L). For raw data, please refer to the S1 Data file.

Therefore, we next determined whether S1pr2 signalling was necessary to down-regulate Notch signalling. Notch levels normally drop, concurrently with differentiation, on 14.5 dpc explants maintained for just 1 d in culture, as shown by Notch intracellular domain (NICD) and Hes-1 immunofluorescence (Fig 8B, 8C, 8E and 8F). Notch down-regulation failed to take place in explants with S1pr2 signalling block (Fig 8H and 8I) but was restored by the addition of 20 μM of S1p (S8 Fig), further establishing the down-regulation of Notch signalling by S1p. Because transcript levels of Notch signalling components did not point to an overall transcriptional regulation of Notch by S1pr2 signalling (S1 Table), we investigated whether such regulation may occur posttranscriptionally. Sel-1 suppressor of lin-12-like (Sel1l) is a member of the endoplasmic reticulum (ER)-associated protein degradation (ERAD) pathway, first identified in *Caenorhabditis elegans* as a negative posttranscriptional regulator of Notch signalling [62,63]. *Sel1l* is expressed in the epithelium of the developing pancreas [64] (Fig 8A), and its disruption resulted in loss of endocrine and acinar differentiation and prolongation of the progenitor cell state [65], a phenotype strikingly similar to that of the S1pr2 signalling block. Consistent with a role in differentiation, Sel1l is up-regulated in differentiating pancreas explants (Fig 8D). S1pr2 signalling block resulted in the loss of the Sel1l protein, concomitant with maintenance of Notch signalling (Fig 8G). Further confirming this finding, activation of the Notch signalling above the endogenous levels with the addition of 10 μM of DSL resulted in a strong decrease of Se1l1 levels (S8 Fig). Moreover, addition of 20 μM of S1p restored Se1l1 levels, confirming its dependence upon S1p signalling (S8 Fig). Se1l1 protein levels were also restored in the absence of S1pr2 signalling by 1 μM of the specific proteasome inhibitor MG132 (Fig 8J), and this was sufficient to restore attenuation of Notch signalling (Fig 8K and 8L). Therefore, S1pr2 signalling stabilises the Sel1l protein that in turn mediates the Notch signalling down-regulation that is necessary for acinar and endocrine differentiation.

These results suggested that S1pr2 signalling mediates Notch down-regulation to induce endocrine and acinar differentiation but did not clarify whether this was sufficient and whether Notch signalling affected YAP levels. To answer these questions, we first blocked Notch signalling without affecting endogenous S1pr2 signalling using 10 μM of the gamma-secretase inhibitor DAPT in ALI cultures of 14.5 dpc pancreata [19,66]. This induced a large increase in the number of Ngn3^+^ cells and resulted in preferential endocrine differentiation (Fig 7B and 7D) (compare Fig 4A, 4B, 4E and 4F with Fig 7E and 7F and compare S4 Fig with S8 Fig, see also Fig 7C).

We then addressed whether Notch down-regulation in the absence of S1pr2 signalling would be sufficient to initiate and maintain differentiation. We found that despite the large number of Ngn3^+^ cells at 14.5 dpc + 2 d explants (Fig 7B and 7G) and the transient initiation of differentiation (compare S4 Fig with S8 Fig), neither endocrine nor acinar cell differentiation were maintained (Fig 7F and 7I), suggesting that Notch down-regulation is not sufficient but acts in concert with other effectors of S1pr2 signalling. We have shown above that with regard to endocrine specification YAP plays an important role. Importantly, we have further found that YAP levels depend upon S1pr2 but not Notch signalling. YAP levels did not change when Notch signalling alone was inhibited or when Notch signalling was inhibited concurrently with S1pr2 inhibition (Fig 7A). This suggested that YAP levels are regulated by S1pr2 signalling through the G_αi_ function and not Notch attenuation (Fig 6G).

Taken together, these experiments showed that S1pr2 signalling attenuates Notch activity through the protein stabilisation of its negative regulator Sel1l. Both this attenuation and the YAP stabilisation are necessary for the specification of the endocrine and acinar lineages.

## Discussion

During development, cell fate decisions need to be coupled to the implementation of differentiation programs so that different cell types in each organ are generated in the appropriate numbers. Notch signalling is widely employed as a regulator of binary cell fate decisions, and it also plays a key role in sequential lineage segregation during pancreas development [9–12,14,18,19]. How Notch activity is coordinated with the implementation of specification programs is not well understood. Furthermore, signalling molecules that trigger lineage differentiation remain largely elusive. Our findings revealed the implication of the S1p signalling pathway in key pancreas developmental decisions. We found that the S1p signalling pathway has a dual role in pancreas development by regulating lineage allocation and differentiation and thus coordinating these processes.

Consistent with the notion that S1p is an important signalling molecule for pancreas development, expression of the S1p generating enzymes at both the protein and the mRNA levels is substantially higher in the epithelium and gradually increases during the secondary transition. We found that S1pr2 is the main S1p receptor expressed in the developing epithelium. Development of the acinar and endocrine lineages was severely delayed in *S1pr2* null embryos, but specific up-regulation of the functionally related receptors *S1pr3* and *Lpar1* in the epithelium compensated and eventually restored pancreas development. Acute S1pr2 blocking in wt explants prevented this compensation, resulting in complete loss of the endocrine and acinar lineages and a massive appearance of duct-like cells. Down-regulation of *Sox9* expression may contribute to the loss of endocrine and acinar lineages [67]. The remaining cells are clearly of ductal identity, although ductal gene expression is perturbed consistent with the interdependence of pancreatic lineage development [22].

**Fig 9:**
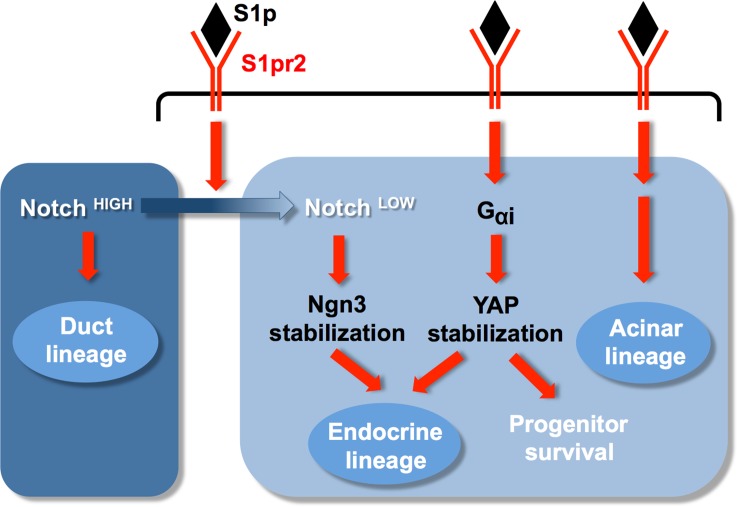
S1pr2 signalling regulates pancreatic lineage allocation and specification. S1pr2 signalling attenuates Notch signalling to promote the segregation of tip MPCs that will give rise to acinar progenitors and bipotent trunk progenitors and, in the latter, to promote the generation of endocrine progenitors via Ngn3 stabilisation. These are two temporally distinct steps shown here as one for simplicity. Ngn3 is stabilised post-translationally in Notch^low^ cells [19]. Additionally, S1p signalling stabilises YAP to enable progenitor survival. G_αi_ activation mediates the stabilisation of the YAP protein to enable endocrine specification. Both YAP stabilisation and Notch down-regulation are necessary for endocrine specification.

This experimental set up provided us with a means to unravel the downstream events. Specific blocking of the S1pr2 downstream effector G_αi_, either in explants or genetically, dramatically and selectively reduced endocrine lineage specification, suggesting that different Ga subunits mediate distinct aspects of pancreas development that could be exploited in stem cell differentiation protocols. Loss of S1pr2 signalling or G_αi_ function resulted in destabilisation of the YAP transcriptional coactivator. Inactivation of the Hippo pathway by pancreas selective disruption of the Mst1/2 kinases led to increased levels of YAP in acinar cells and their subsequent ductal metaplastic conversion [68,69]. However, the role of YAP during pancreas development has not been genetically explored. Genetic temporal inactivation of *YAP* specifically reduced endocrine specification without affecting the appearance of Ngn3^+^ cells. YAP stabilisation by proteasome inhibition in the absence of S1pr2 signalling restored endocrine specification. Furthermore, additional S1p signalling could not restore endocrine specification levels in the *YAP*-deleted pancreata, establishing that an S1p to S1pr2, G_αi_, and YAP stabilisation cascade is necessary for the endocrine differentiation program to be triggered (Fig 9). YAP mRNA levels are significantly higher in the epithelium, and these findings explain the selective importance of the S1p signalling/YAP axis for the development of the epithelium.

S1p signalling block was accompanied by cell death and down-regulation of *CTGF*, a secreted survival factor and known YAP target gene [47–49]. Exogenous addition of CTGF in S1pr2 blocked pancreata rescued the cell death but not the specification defects underlining distinct functions of S1p signalling in progenitor survival and specification (Fig 9).

Several effects of S1pr2 signalling loss would be consistent with maintenance of high Notch activity, raising the possibility that S1p signalling regulates Notch. We found that S1p signalling is necessary for the maintenance of Sel1l protein levels, a member of ERAD pathway, and negative Notch regulator [62,63]. *Sel1l* deficiency impaired endocrine and acinar differentiation and maintained the progenitor cell state [65]. Consistent with that, S1p signalling attenuates Notch activity, a necessary step to trigger differentiation of the acinar and endocrine lineages [9–12,14,18,19]. Rescue of the Sel1l protein levels by proteasome inhibition restored Notch down-regulation in the absence of S1pr2 signalling. However, Notch down-regulation on its own was not sufficient to sustain endocrine and acinar differentiation in the absence of S1pr2 signalling, demonstrating that another effector of the latter is also necessary for endocrine specification, and we have shown that this effector is G_αi_, which mediates YAP stabilisation (Fig 9). Interestingly, Ngn3 protein levels are dependent upon Notch signalling but not on YAP, suggesting that S1p-dependent YAP stabilisation may be acting as a primer of the endocrine program that is then realised in the cells that stabilise Ngn3 protein levels.

Developmental pathways are often employed in adult tissues during regeneration, and their application has also been pivotal in the generation of glucose responsive beta-like cells from human pluripotent stem (PS) cells [70–72]. During pancreas development, TEAD and its coactivator YAP activate key pancreatic signalling mediators and transcription factors to regulate the expansion of pancreatic MPCs that will generate all the pancreatic lineages. After the onset of differentiation, YAP is rapidly degraded in acinar and endocrine cells but is retained in ductal cells [13,68,69]. Our findings suggested an additional role for YAP in activating the endocrine differentiation program and identified S1p as an important signal that maintains its stability through the secondary transition.

This work identified a new signalling pathway in pancreas development that leads to YAP stabilisation and Notch attenuation in the developing pancreas, thus linking lineage allocation and differentiation with a molecular player central for tissue growth control. These findings have important implications for the efficient generation of endocrine cells from pluripotent stem cells and for pancreas regeneration.

## Materials and methods

### Ethics statement

Animal maintenance and experimentation were conducted in accordance with the FELASA recommendations and the ethical and practical guidelines for the care and use of laboratory animals set by the competent veterinary authorities in the authors’ institutions.

### Mouse strains, maintenance, and genotyping

Animal maintenance and experimentation were conducted in compliance with the FELASA recommendations and the ethical and practical guidelines for the care and use of laboratory animals set by the competent veterinary committees in the author’s institutions. Mouse mutant and transgenic lines used were *S1P2*^*tm2Ytak*^ [73], *YAP*^*fl/fl*^ [74], *Tg*^*Pdx1CreERT2*^ [57], *S1pr2*^*tm1Rlp*^ [29], and *Gt(ROSA)26Sor*^*tm1(ptxA)Cgh*^ [38] purchased from the Mutant Mouse Regional Resource Center (MMRRC; Chapel Hill, NC) and *Gt(ROSA)26Sor*^*tm9(CAG-tdTomato)hZE*^ [75] and *Tg*^*(Pdx1-cre)6Tuv*^ [76] purchased from The Jackson Laboratory (Bar Harbor, Maine) (S3 Table). All mouse strains used were interbred onto the same genetic background (C57BL/6J). Genotyping was performed by conventional PCR on genomic DNA isolated from mouse-tails using standard procedures. Briefly, mouse tails were dissolved in Tail buffer (100 mM Tris-HCl pH 8.0, 200 mM NaCl, 5mM EDTA, and 0.2% SDS) with 50 μg/ml Proteinase K (Sigma) overnight at 55°C. Following a protein extraction step with Phenol/Chloroform (Sigma), genomic DNA was precipitated from the aqueous phase with 100% ethanol and finally resuspended in TE buffer (10 mM Tris-HCl pH 8.0 and 1 mM EDTA). PCR conditions and primers used are provided in S4 Table.

### Mouse injections

Tamoxifen (Sigma) was diluted in corn oil and injected ip in pregnant dams for 6 consecutive days starting at 10.5 dpc with 1 mg, followed by 0.5 mg in the following days. To ensure homogeneity in the analysis, all newborns were collected either following delivery or by caesarean section at 19.5 dpc (P1). JTE013 was diluted in PBS and injected ip at 2 or 5 mg/Kg body weight in pregnant dams at 13.5, 14.5, and 15.5 dpc. Embryos were collected at 16.5 dpc for analysis.

### Organotypic cultures of embryonic pancreata

Dorsal pancreatic buds were dissected under a light stereoscope (Leica MZ75) at 13.5 or 14.5 dpc and cultured for 1, 2, 3, or 6 d on 0.4-μm pore diameter filters (Millicell-CM; Millipore) in DMEM (Gibco) supplemented with N2 (Gibco) and streptomycine-penicillin-glutamine (Gibco). Treatments were with JTE013 (Tocris) at 15 μM, CTGF (Peprotech) at 50 ng/ml, PTX (Calbiochem) at 10 μg/ml, DSL (CDDYYYGFGCNKFCRPR)(AnaSpec, AS-61298) at 10 μM, DAPT (Sigma) at 10 μM, C3 exoenzyme (Biomol) at 5 μg/ml, GP antagonist-2A (Enzo) at 50 μM, VPC23019 (Avanti Lipids) at 50 μM, Ki16425 (Sigma) at 20 μM, MG132 (Sigma) at 1 μM and S1P (Tocris) at 20 μM. Controls were treated with the same amount of solvent where appropriate. Chemicals were replenished with each medium change daily for 2-day and 3-day cultures or every second day for 6-day cultures.

### Immunostainings on cryosections

Dissected pancreata were fixed in 4% paraformaldehyde (PFA; Sigma), washed in PBS, and dehydrated in 30% sucrose (Sigma) overnight at 4°C. Tissues were embedded in optimal cutting temperature (OCT) compound (BDH), cut into sections of 12-μm thickness and mounted onto Superfrost slides (VWR) for storage at −80°C. For immunostainings, cryosections were post-fixed in 4% PFA for 5 min and blocked for 1 h at room temperature in 10xBlocking solution (10% goat serum, 0.1% BSA, and 0.3% Triton X-100 in PBS). Primary antibodies were diluted in 1xBlocking solution and incubated o/n at 4°C, whereas secondary antibodies, also diluted in 1xBlocking solution, were incubated for 2 h at room temperature. All washes were done in PBST (PBS with 0.3% Triton X-100). After the final washes, slides were mounted with DAPI (Vectashield; Vector or Prolong Gold antifade reagent with DAPI; Molecular Probes) and taken for fluorescent microscopy.

### Immunostainings on paraffin sections

Dissected pancreata were fixed in 10% formalin (Sigma) for 1 h at 4°C, washed in PBS, and taken into 70% ethanol. Tissues were then gradually dehydrated in a series of ethanol concentrations (70%, 95%, and 100% for 10 min each), before transferring in xylene for 10 min and then into liquid paraffin for an additional 10 min. Sections of 6-μm thickness were cut on a Leica RM2265 microtome and mounted onto polylysine-treated slides (VWR). For immunofluorescence, slides were incubated with Xylene for 20 min for deparafinisation and then immersed in a reverse series of ethanol concentrations (100%, 95%, 70%, and 50%) for rehydration followed by washes in PBS. Sections were then processed for immunofluorescence as described for cryosections, with the addition of an antigen-retrieval and a signal-amplification step. For antigen retrieval, slides were immersed in 10 mM Sodium citrate pH 6.0, boiled in a microwave oven at 600 W for 4 min, and then left to simmer at 250 W for 15 min. Slides were allowed to cool at room temperature for 40 min and then washed in distilled water and finally in PBS before proceeding with blocking. For amplification, a biotinylated secondary antibody (Vector) was used, followed by incubation with an avidin/biotin-HRP enzyme complex (ABC kit; Vector) for 1 h at room temperature. Slides were then washed in PBST and incubated with Tyramide-Cy3 diluted at 1:50 in TSA buffer (TSA kit; Perkin Elmer) for 3 min at room temperature to achieve HRP signal amplification. After a final wash in PBST, slides were mounted with DAPI (Vectashield) and taken for fluorescent microscopy.

### Morphometric analysis

Morphometric analysis on embryonic pancreata and pancreatic explants was performed using immunofluorescent images taken at saturation and the ImageJ (Fiji) software. More specifically, the “Analyse Particles” tool of ImageJ was used to quantitate the number of pixels that correspond to signal area on an immunofluorescent image. For the 14.5 dpc pancreata and all pancreatic explants, analysis was performed using at least three 12-μm-thick cryosections per sample 24 μm apart, spanning the entire tissue. For the 19.5 dpc and P1 pancreata, analysis was performed using at least six 12-μm-thick cryosections per sample, 120 μm apart, spanning the entire tissue. For endocrine, acinar, and ductal cell quantitation, the signal area of C-peptide/Glucagon-, amylase-, and Cytokeratin19-positive immunofluorescence, respectively, was measured and divided by the corresponding total signal area for DAPI, thus normalising for mass. The same procedure was followed for Pdx1-, Ptf1a-, Nkx6.1, and YAP-signal area quantitation. For quantitation of epithelial mitotic activity, PH3-positive nuclei were counted and divided over the DAPI area of the epithelium as marked by E-cadherin co-staining. Similarly, for endocrine progenitor quantitation, Ngn3-positive nuclei were counted and divided over total DAPI area. To quantify the recombination efficiency, double Tdt + each marker (Cpeptide+Glucagon, Amylase, CK-19) positive cells were counted and this number divided for the total number of cells expressing each marker. For each quantitation, at least three pancreata of each genotype and/or condition were analysed.

### RNA isolation and real-time PCR

Total RNA was prepared using the RNeasy kit with on-column genomic DNA digestion following the manufacturer’s instructions (Qiagen). First strand cDNA was prepared using Superscript II RT (Invitrogen). Real-time PCR primers were designed using the Primer 3 software (SimGene), specificity was ensured by in silico PCR, reactions were performed with SYBR-Greener (Invitrogen) using an ABI PRISM 7000 machine or a Roche LC480 machine, and primary results were analysed using the machine’s software. Reactions were carried out from at least four independent samples with the exception of embryonic pancreata from JTE013 injected dams where three samples were used. Absolute expression values were calculated using the ΔCt method using *β-actin* for normalisation except for the embryonic pancreata from JTE013 injected dams where Eif3 was used. Primers used were further evaluated by inspection of the dissociation curve. Primer sequences were as follows:

*S1pr2* F: CACTACAATTACACCAAGGAGAC, R: CAGCACAAGATGATGATGAAGG;

*Sphk1* F: AGAAGGGCAAGCATATGGAA, R: ACCATCAGCTCTCCATCCAC;

*Sphk2* F: ACTGCTCGCTTCTTCTCTGC, R: GCCACTGACAGGAAGGAAAA;

*Lpar1* F: TCTTCTGGGCCATTTTCAAC, R: TCCTGGGTCCAGAACTATGC;

*S1pr3* F: GGGAGGGCAGTATGTTCGTA, R: GGCATCATATGGCCTCATCT;

*Yap1* F: GCGGTTGAAACAACAGGAAT, R: TGCTCCAGTGTAGGCAACTG;

*Pdx1* F: TCCACCACCACCTTCCAG, R: CAGGCTCGGTTCCATTCG;

*Ctgf* F: AGCGTCCAGACACCAACCT, R: GGTAGGAGGATGCACAGCAG;

*β-actin* F: TGGCTCCTAGCACCATGA, R: CCACCGATCCACACAGAG

*Eif3* F: TCTCCGGCCGTACCGGCTAA, R: GAGCTGGCGTGGATGGGGTG

### RNA sequencing and bioinformatic analysis

Embryonic pancreata (13.5, 14.5, or 15.5 dpc) from up to three pups of the same genotype were pooled to generate one sample. For ALI cultures, up to three pancreata having undergone the same treatment (normal medium, JTE013 or JTE013, and CTGF supplemented) were pooled to generate one sample. Three independent samples from each stage/genotype or ALI condition were used as biological replicates.

Total RNA was prepared using the RNeasy kit with on-column genomic DNA digestion (Qiagen), and only RNA with an integrity number of ≥8 was used. mRNA was isolated from 1 ug total RNA by poly-dT enrichment using the NEBNext Poly(A) mRNA Magnetic Isolation Module according to the manufacturer’s instructions. Final elution was done in 15 ul 2x first strand cDNA synthesis buffer (NEBnext, NEB). After chemical fragmentation by incubating for 15 min at 94°C, the sample was directly subjected to the workflow for strand-specific RNA-Seq library preparation (Ultra Directional RNA Library Prep, NEB). For ligation, custom adaptors were used (Adaptor-Oligo 1: 5'-ACA-CTC-TTT-CCC-TAC-ACG-ACG-CTC-TTC-CGA-TCT-3', Adaptor-Oligo 2: 5'-P-GAT-CGG-AAG-AGC-ACA-CGT-CTG-AAC-TCC-AGT-CAC-3'). After ligation, adapters were depleted by an XP bead purification (Beckman Coulter) adding bead in a ratio of 1:1. Indexing was done during the following PCR enrichment (15 cycles) using custom amplification primers carrying the index sequence indicated with ‘NNNNNN’. (Primer1: Oligo_Seq AATGATACGGCGACCACCGAGATCTACACTCTTTCCCTACACGACGCTCTTCCGATCT, primer2: GTGACTGGAGTTCAGACGTGTGCTCTTCCGATCT, primer3: CAAGCAGAAGACGGCATACGAGAT NNNNNN GTGACTGGAGTT). After two more XP beads purifications (1:1), libraries were quantified using Qubit dsDNA HS Assay Kit (Invitrogen). Libraries were equimolarly pooled and sequenced on an Illumina HiSeq 2500, resulting in ca. 16–20 million single end reads per library.

After sequencing, FastQC (http://www.bioinformatics.babraham.ac.uk/) was used to perform a basic quality control on the resulting reads. As an additional control, library diversity was assessed by redundancy investigation in the aligned reads. Alignment of the short reads to the mm10 reference was done with GSNAP (v 2014-12-17) [77], and Ensembl gene annotation version 69 was used to detect splice sites. The uniquely aligned reads were counted with featureCounts (v1.4.6) [74] and the same Ensembl annotation.

Normalisation of the raw read counts based on the library size and testing for differential expression between the different conditions was performed with the DESeq2 R package (v1.6.2) [78]. To calculate the number of regulated genes between two conditions, we accepted a maximum of 10% false discoveries (padj ≤ 0.1) and considered only genes with normalised counts >100 in either condition and fold change <0.6 or >1.6. Unknown transcripts, pseudogenes, and RNAs other than mRNAs were not considered.

For the PCA, the raw read counts of the conditions to be analysed and their replicates were transformed with the variance stabilising transformation of the DESeq2 package. The top 500 genes that showed the highest variance were selected and used for the PCA calculation.

### TUNEL assays

TUNEL assays on cryosections were performed using a fluorescent cell death detection kit (Roche) according to the manufacturer’s instructions. Briefly, cryosections were washed in PBS and incubated in Permeabilisation solution (0.1% Triton X-100 in 0.1% Na-citrate) for 2 min at 4°C. For labelling of DNA strand breaks, slides were incubated with 50 μl of TUNEL reaction mixture (fluorescein-labelled nucleotides mixed with terminal deoxynucleotidyl transferase) for 1 h at 37°C. Slides were finally rinsed in PBS, mounted with DAPI (Vectashield), and analysed by fluorescent microscopy. Slides that were incubated with DNase I prior to the labelling reaction were used as a positive control.

### Western blotting

Protein extraction, gel electrophoresis, and immunoblotting procedures were according to standard protocols. For protein extraction, samples were lysed in RIPA buffer (50 mM Tris-HCl pH 7.4, 1% NP-40, 0.5% Na-deoxycholate, 0.1% SDS, 150 mM NaCl, and 2 mM EDTA) and supplemented with protease and phosphatase inhibitor cocktails (Sigma). Proteins were loaded at 30 μg/lane on a polyacrylamide gel for electrophoresis, and protein transfer was subsequently performed on a standard nitrocellulose membrane (Amersham). Blocking was performed in 5% milk in TBST (50 mM Tris-HCl pH 7.6, 150 mM NaCl, and 0.05% Tween-20) for 1 h at room temperature, and primary and secondary antibodies were diluted in 1% milk in TBST and incubated overnight at 4°C or for 2 h at room temperature, respectively. Signal was developed using the ECL kit (Perkin Elmer) according to the manufacturer’s instructions. Primary antibodies used were rabbit anti-Yap1 (1:1000; Proteintech), rabbit anti-S1P2 (1:500; Thermo Fisher Scientific), rat anti-E-Cadherin (1:2,000; Zymed), and mouse anti β-actin (1:5,000; Santa Cruz). Secondary antibodies were anti-rabbit, anti-rat, and anti-mouse horseradish peroxidase (HRP)-conjugated goat antibodies (1:5,000; Dako).

### Fluorescence activated cell sorting

The epithelial and mesenchymal components of embryonic and cultured pancreata were separated by FACS based on the epithelial expression of Cd49f as described [79]. Briefly, pancreata were dissected and incubated in a 0.05% Trypsin/EDTA solution (Gibco) for 5 min at 37°C. The reaction was terminated with 10% FBS (Sigma), tissues were dispersed with mild pipetting, and single cells were collected by centrifugation (400 g; 4 min). Cells were resuspended in PBS with 0.5% FBS, and after blocking with rat IgG (Abcam) for 15 min, cells were incubated with a Cd49f-FITC (BD Pharmigen) antibody, added at 1:50 dillution for 1 h on ice with gentle rocking. Cells were collected by centrifugation, resuspended in PBS with 0.5% FBS, and sorted by FITC fluorescence intensity on a BD FACS Aria III, following standard procedures [79]. Two fractions were collected consisting of fluorescent (epithelial) and non-fluorescent (mesenchymal) cells, which were subsequently processed for RNA isolation and real-time PCR analysis. The efficiency of the separation was independently confirmed by determining Pdx1 expression in the two fractions.

### X-gal stainings

Cryosections of embryonic pancreata were washed in PBS and incubated with 1 mg/ml 5-bromo-4-chloro-3-indolyl-β-D-galactopyranoside (X-Gal; Roche) in staining solution [5mM K_3_Fe(CN)_6_, 5mM K_4_Fe(CN)_6_, 2 mM MgCI_2_, and 5 mM EGTA] for 8 h at 37°C. For double stainings, X-gal–treated sections were rinsed in PBS, incubated in blocking solution (10% normal goat serum, 0.3% Triton X-100) for 1 h at room temperature, and subsequently processed for immunofluorescence as described. For imaging, X-gal stainings were photographed in bright field, and the image was inverted and assigned to the green colour of the RGB composite. This was then merged with the immunofluorescent image (in red) and the DAPI image (in blue) of the corresponding field photographed under UV.

### Statistical analysis

Statistical significance in morphometric analyses and real-time PCRs was determined by the Student’s *t* test for two-tailed distributions of unpaired groups. Error bars represent the standard error of the mean (SEM). *p* < 0.05 was considered significant.

### List of antibodies used for immunofluorescence

Primary antibodies used were rabbit anti-Ngn3 (1:100; Acris), rat anti-E-cadherin (1:400; Zymed), rabbit anti-C-peptide (1:200; Linco), rabbit anti-Amylase (1:300; Sigma), rabbit anti-Ptf1a (1:3,000; Gift from B. Breant), rat anti-Cytokeratin19 (1:250; DSHB), mouse anti-Glucagon (1:500; Sigma), rabbit anti-Pdx1 (1:5,000; Gift from C. Wright), mouse anti-Pdx1 (1:250; DSHB), rabbit anti-Sphk (1:250; Abcam), mouse anti-Nkx6-1 (1:1,000; DSHB), mouse anti-Insulin (1:1,000; Sigma), rabbit anti-PH3 (1:500; Cell Signaling), rabbit anti-Yap1 (1:200; Cell Signaling), rabbit anti-NICD (1:100; Cell Signaling), rat anti-Hes1 (1:500; MBL-Biozol), and rabbit anti-Sel1l (1:250; Abcam). Secondary antibodies were anti-mouse, anti-rabbit, and anti-rat Alexa-488-, Alexa-568-, and Alexa-633-conjugated goat antibodies (1:500; Molecular Probes), as well as anti-rabbit and anti-rat biotinylated goat antibodies (1:100; Vector). For Nkx6.1 and Pdx1 mouse antibody staining together with Sphk, a Goat anti-Mouse IgG affiniPure Fab Fragment from Jackson ImmunoResearch (115-007-003) was used at 1:50 dilution following the instructions of the manufacturer.

## Supporting information

S1 Fig *S1pr2* and *Sphk* expression during pancreas development. (A-F) Tracking β-galactosidase activity with the X-gal assay from heterozygous *S1P2^tm1lacz^* mouse embryos revealed that *S1pr2* was expressed in the pancreas at 9.5 dpc (A, D) and was mainly detected in the mesenchyme until 12.5 dpc because immunofluorescence showed that *S1pr2 *expression was apparent in a few Pdx1^+^ (B, C) and E-cadherin^+^ (E, F) epithelial cells. (G-I, Q) Epithelial *S1pr2* expression detected by the x-gal activity of the modified allele in heterozygous *S1P2^tm1lacz^* mouse embryos peaked at 14.5 dpc, and immunofluorescence showed that it was co-locoalized with Nkx6.1^+^ trunk cells (eg arrows in G) as well as with Ptf1a^+^ tip cells (eg arrows in H). The same analysis showed that by 16.5 dpc, *S1pr2* expression was eliminated in nascent endocrine C-Pep^+^ and Gcg^+^ (Endo) cells and significantly reduced in the rest of the tissue (I). At P1 expression had disappeared completely (Q).(J-L) Quantitative PCR analysis on FACS-isolated epithelial and mesenchymal components of the developing pancreas at 13.5, 14.5 and 15.5 dpc confirmed that *S1pr2*, *Sphk1* and *Sphk2* expression peaked at 14.5 dpc (K, L), and that *Sphk1* and *Sphk2* expression was predominantly epithelial (L). The efficiency of the separation was confirmed independently by determining *Pdx1 *expression by qPCR in the mesenchymal and epithelial components of FACS separated cells (J). (M-P) Time course of Sphk expression by immunofluorescence at 12.5, 14.5 and 16.5 dpc confirmed the presence of the protein primarily in the epithelium (M-O) and co-localization with Nkx6-1 at 14.5 dpc in the epithelial trunk cells (P). Scale bars, 40μm (A, F), 80μm (G-I, P), 10μm (M-O), 160μm (Q); *p< 0.05, **p<0.01, ***p<0.001, ns not significant in reference to epithelial expression in the corresponding time point (J, L) or corresponding to the E14.5 samples (K); Error bars show SEM. For raw data please refer to the S2 Data file.
(TIF)

S2 Fig Analysis of progenitor marker genes, proliferation and expression of *Lpar1* and *S1pr3* in the *S1pr2 ^tm1Rlp^* null embryonic pancreata and postnatal endocrine phenotype of *S1pr2^tm1Rl^* null mice. (A-G) RNA Seq gene expression profiling revealed that transcription factors and other genes implicated in epithelial progenitor specification and maintenance were not significantly affected in *S1pr2^tm1Rlp^* null pancreata at 14.5 dpc (A). RNA seq counts of both *S1pr3* and *Lpar1* were increased in the *S1pr2^tm1Rlp^*null pancreata at 14.5 dpc (B). Abrogating S1pr2 signalling by intraperitoneally injecting pregnant mice with 2 or 5 mg/kg of body weight JTE013 at 13.5, 14.5 and 15.5 dpc resulted in upregulation of both *S1pr3* and *Lpar1* at 16.5 dpc as shown by qPCR (C). Quantitative PCR analysis on isolated epithelial and mesenchymal components of the wt developing pancreas at 13.5, 14.5 and 15.5 dpc showed that expression of expression of both *S1pr3* (D) and *Lpar1* (E) was predominantly mesenchymal. Quantitative PCR for *Pdx1* expression was used to confirm the efficiency of mesenchymal and epithelial separation by FACS (F). Immunofluorescence and quantitation of the ratio pH3^+^ / E-cadherin^+^ cells in wt and *S1pr2* null pancreata showed that epithelial proliferation was not affected (G). (H) Western blot analysis on wild-type and *S1pr2^tm1Rlp^* null pancreata at 14.5 dpc show that S1Pr2 protein is completely absent in the *S1pr2^tm1Rlp^*null. (I-K) The number of endocrine cells (C-pep^+^ and Gcg^+^) in *S1pr2* null newborns is similar to wt littermates (I) and 8 week *S1pr2* null adults show no difference in fasting glucose blood levels (J) or in glucose tolerance test (K). *padj* < 0.05 (B); *p< 0.05, **p<0.01, ***p<0.001, ns not significant in reference to expression in untreated or control samples; Error bars show SEM. For raw data please refer to the S2 Data file.
(TIF)

S3 Fig *S1pr2^tm1Rlp^* null embryonic pancreata in ALI cultures showed defects in lineage specification; S1p rescues endocrine specification in JTE013-treated pancreata in ALI cultures. (A-I, M, N) Immunofluorescence analysis showed that 14.5 dpc *S1pr2* null pancreata in ALI cultures for 6 days (14.5 dpc + 6ds) gave a strongly reduced number of C-pep^+^ and Gcg^+^ endocrine cells (B, E, N), a strongly reduced number of Amy^+^ acinar cells and an increased number of CK19^+^ duct-like cells (C, F, N). S1pr2 block by 15μM JTE013 in 14.5 dpc + 6ds ALI cultures of wild-type pancreata resulted in morphological defects characterised by an absence of the dense cell clusters observed by brightfield microscopy in untreated wt and *S1pr2^ tm1Rlp^* null cultures (compare M to A and D). In contrast, 14.5 dpc + 6ds ALI cultures of *S1pr2* null pancreata in the presence of 15μM JTE013 caused no such morphological defects (G), and immunofluorescence analysis showed that it did not further affect specification of endocrine (C-peptide^+^ and Glucagon^+^)(B, E, H, N), acinar (Amylase^+^) or ductal (CK19^+^) (C, F, I, N) cells confirming the specificity of JTE013 for S1pr2 also in this context. (J-L) Immunofluorescence analysis showed that the presence of 20 μM S1p rescued specification of endocrine (Cpep^+^ and Gcg^+^) cells in JTE013-treated 14.5 dpc + 6 ds ALI cultures (K), and to a lesser extent specification of acinar (Amy^+^) and ductal (CK19^+^) cells (L). Morphological defects were also rescued under these conditions as evidenced by brightfield microscopy (J). Quantitations are provided in S7A Fig.Scale bars, 80μm (B, C, E, F, H, I, K, L) and 100μm (A, D, G, J, M); ***p<0.001, in reference to corresponding untreteated controls; Error bars show SD. For raw data please refer to the S2 Data file.
(TIF)

S4 Fig S1pr2 block in pancreatic ALI cultures affects lineage specification. (A-I) Immunofluorescence showed that terminally differentiated endocrine (C-pep^+^ and Gcg^+^), acinar (Amy^+^) and ductal (CK19^+^) cells are scarce in wt 14.5 dpc embryonic pancreata (A, B), but are readily detected after 2 days in ALI cultures (C, D). S1pr2 block with 15 μM JTE013 in 14.5 dpc + 2 ds ALI cultures and immunofluorescence analysis showed a loss of endocrine (C-pep^+^) and acinar (Amy^+^) cells (C-F) and a decrease in epithelial proliferation (ratio of pH3^+^/E-Cad^+^ cells) (G). In these experiments a small increase in cell death was evident by TUNEL analysis (H, I). (J, K) High-magnification immunofluorescence showing the presence of Ptf1a^+^/Pdx1^-^ cells in 14.5 + 2 ds ALI cultures (arrows in J), which are lost upon S1pr2 block (K). (L, M) RNA Seq gene expression analysis on 14.5 dpc + 2 ds pancreatic ALI cultures, revealed that expression of progenitor markers was only weakly affected upon S1pr2 block with 15 μM JTE013, with the notable exception of *Nkx6*.*2* and *Dll1* (L). Venn diagram for up- and down-regulated genes in 14.5 dpc *S1pr2^ tm1Rlp^* null pancreata and wt pancreata and pancreata cultured for 2 days in standard conditions or with S1pr2 signaling blocked by 15 μM JTE013 (M). Scale bars, 80μm (A-F, I, J), 25μm (J, K). For raw data please refer to the S2 Data file.
(TIF)

S5 Fig CTGF rescues cell death caused by S1pr2 block. (A-C) Quantitative PCR analysis showed that *CTGF *expression peaks at 14.5 dpc (A) and that it was expressed in both the mesenchyme and the epithelium of 13.5, 14.5 and 15.5 dpc embryonic pancreata (B). S1pr2 block in 14.5 dpc + 2 ds ALI pancreatic cultures with 15 μM JTE013 causes a 3.5-fold decrease in *CTGF* expression as shown by RNA Seq (C). (D-I) Immunofluorescence analysis of 14.5 dpc + 2 ds (D-F) or 14.5 dpc + 6 ds (G-I) ALI cultures that were S1pr2 signaling blocked with 15 μM JTE013 and supplemented with 50 ng/ml CTGF. Addition of CTGF was not sufficient to restore the number of Ngn3^+^ cells (D), there was no effect on the expression pattern of Pdx1 and Ptf1a progenitor markers (E) and epithelial proliferation remained decreased (F). S1pr2 block in 14.5 dpc + 6 ds ALI cultures eliminated Nkx6.1^+^ cells (G, H) but supplementation with CTGF for the duration of the culture lead to an expanded population of Nkx6.1^+^ cells (I) (J-N) Comparison of gene expression changes detected by RNA Seq analysis in 14.5 dpc + 2 ds dpc ALI cultures under conditions of S1pr2 block (15 μM JTE013), in the presence (grey bars) or absence (black bars) of CTGF. Gene expression changes (fold regulation) of progenitor (K), endocrine (J), acinar (L) and duct (M) markers were strikingly similar in the two conditions as compared to untreated 14.5 dpc + 2 ds ALI cultures. Principal component analysis (PCA) showed that S1pr2-blocked explants with or without CTGF cluster much closer together than with untreated control explants (N). (O) Quantitative PCR analysis showed that the presence of 20μM S1p in S1pr2-blocked 14.5 dpc + 2 ds ALI cultures restored CTGF expression to control levels. Scale bars, 80μm (D, E, G-I); *padj*<0.05 (C); *p< 0.05, **p<0.01, ***p<0.001, ns not significant in reference to E14.5 samples (A) or untreated samples (F); Error bars show SEM. For raw data please refer to the S2 Data file.
(TIF)

S6 Fig Block of G_αi_ subunits does not affect the acinar or ductal lineages. (A-K) Expression of a single allele of the conditional R*OSA26^LSLPTX^* transgene in pancreatic epithelial progenitors using the *Pdx1-Cre* driver *Tg^Pdx1Cre^* resulted in disruption of endocrine cell migration and clustering as shown by immunofluorescence of 14.5 dpc + 6 ds ALI cultures (A, B). Expression of two alleles of the *ROSA26^LSLPTX^* transgene using the same driver did not affect Amy^+^ acinar of CK19^+^ duct cells either at P1 (C, D) or at 14.5 dpc + 6 ds ALI cultures (E, F, J) as shown by immunofluorescence. Similarly, addition of 10 μg/ml of PTX in 14.5 dpc + 6 ds ALI cultures also had no effect on acinar or duct cell specification (K). Immunofluorescence analysis of 14.5 dpc + 6 ds ALI cultures or 14.5 dpc + 2 ALI cultures from *ROSA26^LSLPTX/LSL/PTX^*mice had no defect in endocrine specification and islet clustering (G) or in the generation of Ngn3^+^ cells (H). Quantitation of the effects of G_αi_ block in the number of Ngn3^+^ endocrine progenitor cells showed a strong decrease in both the genetic and ALI culture paradigms (I). (L) Quantitation of the effects of PTX actiivation from the *ROSA26 ^LSLPTX^* transgene using the *Tg^Pdx1Cre^*driver showed a striking loss of C-pep^+^ and Gcg^+^ cells compared to wild-type animals at postnatal day 1 (P1). Scale bars, 80μm (A-H, J, K); ***p<0.001, in reference to untreated ALI cultures (I) or wild-type animals (L); Error bars in I show SEM; Error bars in L show SD. For raw data please refer to the S2 Data file.
(TIF)

S7 Fig Temporal YAP inactivation does not affect acinar or ductal specification. (A) Quantitation of the effects of S1p or the proteasome inhibitor MG132 in S1pr2-blocked 14.5 dpc + 6 ds ALI cultures, on β- (Cpep^+^), acinar (Amy^+^) and duct (CK19^+^) cells. (B) Quantitative PCR analysis in embryonic pancreata at 10.5, 12.5, 14.5 and 15.5 dpc showed that *YAP* transcript levels peak at 14.5 dpc. (C) Quantitative PCR analysis showed that *YAP *is expressed in both the mesenchyme and the epithelium of 13.5 embryonic pancreata but is predominantly expressed in the epithelium at 14.5 and 15.5 dpc pancreata. (D) RNA Seq analysis on 14.5 dpc pancreata and pancreatic ALI cultures showed that YAP gene expression is not affected in S1pr2-nulls and in S1pr2-blocked explants in the presence or absence of CTGF (E) Quantitative PCR analysis showed that *YAP* levels remain unchanged in 14.5 dpc + 2ds ALI cultures treated with 10μg/ml PTX compared to untreated controls. (F) Western blot analysis on 14.5 + 2 ds ALI cultures treated with 50μM of the G_αq_ inhibitor GP-2A or 5μg/ml of the G_α_12/13 inhibitor C3-exoenzyme, showed that YAP levels remained unchanged in both cases, compared to untreated controls.(G-J, N, O) A conditional *ROSA26^LSLtdTomato^*allele was activated using the *Tg^Pdx1CreERT2^* driver and tamoxifen ip injections in pregnant mice. Analysis by immunofluorescence at 14.5 dpc showed that endocrine (C-pep^+^ and Gcg^+^)(G), acinar (Amy^+^) (H) and ductal (CK19^+^) (I) cells were labeled with tdTomato, with a similar efficiency ranging between 38–50% (J). Immunofluorescence analysis of these samples showed that there was no effect on acinar (N) or duct (O) cell specification. (K-M) Immunofluorescence analysis on the above samples at 14.5 dpc indicated that YAP expression in Tg^Pdx1CreERT2^ / YAP^fl/fl^ pancreata (L, M) drops by 64% compared to their wild-type counterparts (K, M). (P-R) However, no differences in the numbers of Ngn3^+^ cells were observed between YAP inactivated (Q,R) and control YAP^fl/fl^ pancreata (P,R). (S-V) Tg^Pdx1CreERT2^ / YAP^fl/fl^ E14.5 pancreata kept in culture for 6 days displayed a 30% reduction in the levels of endocrine cells compared to control YAP^fl/fl^ pancreata explants (S, T, V). Treatment with 20 μM S1P, did not restore the numbers of endocrine cells in the YAP inactivated explants (U, V). Scale bars, 70μm (G-I, P, Q, S-U), 100μm (K, L); ***p<0.001, ns not significant in reference to S1pr2-blocked controls (A), to E14.5 samples (B), to expression in the mesenchymal component of the corresponding time point (C), to untreated control explants (E) or to expresion in control (YAP^fl/fl^) pancreata (N, O, R); Error bars in B, C, E show SEM; Error bars in A, J, M, N, O, R, V show SD. For raw data please refer to the S2 Data file.
(TIF)

S8 Fig Interaction of Notch and S1pr2 signaling in pancreatic ALI cultures. (A-C) Immunofluorecence analysis showed that stimulation of Notch signalling with 10 μM DSL resulted in elimination of Ngn3^+^ cells (A, quantitations in Fig 8B) in 14.5 dpc + 2ds ALI cultures and in a significant reduction in the number of C-pep^+^, Gcg^+^ (B, quantitations in Fig 8C) and Amy^+^ cells in favour of an expanded population of CK19^+^ cells (C, quantitations in Fig 8C) in 14.5 dpc + 6ds ALI cultures (D-F) Immunofluorescence analysis in 14.5 dpc + 1d ALI cultures showed that Sel1l expression was restored in the absence of S1pr2 signalling by 20 μM of S1p (D), and this was sufficient to restore attenuation of both Hes1 (E) and NICD (F) expression. (G-H) Notch inhibition of 14.5 dpc + 2 ds ALI cultures with 10 μM DAPT caused a dramatic transient expansion of C-pep^+^ and Gcg^+^ cells (G), at the expense of Amy^+^ and CK19^+^ cells (H). (I-J) Notch inhibition with 10 μM DAPT together with S1pr2 block with 15 μM JTE013 in 14.5 dpc + 2 ds ALI cultures transiently restored endocrine (C-pep^+^) (I) and acinar (Amy^+^) (J) cell specification. (K-L) Immunofluorescence analysis in 14.5 dpc + 2d ALI cultures showed that Notch stimulation with 10 μM DSL resulted in a significant reduction of Sel1l expression (K, L). Scale bars, 70μm (A-C and G-J), 50μm (D-F, K, L). For raw data please refer to the S2 Data file.
(TIF)

S1 Table Normalised RNA Seq counts, regulation and statistical significance of genes implicated in pancreas progenitors, endocrine, acinar, and ductal cells. (XLSX)

S2 Table Expression of YAP regulated genes. (XLSX)

S3 Table List of mouse lines used. (DOC)

S4 Table Genotyping primers and PCR Conditions. (DOC)

S1 Data Raw data for figures. (XLSX)

S2 Data Raw data for supplementary figures. (XLSX)

## Abbreviations

ADP: adenosine diphosphate
ALI: air–liquid interface
CTGF: connective tissue growth factor
dpc: days post coitum
DSL: Delta-Serrate/lag2
ER: endoplasmic reticulum
ERAD: ER-associated degradation pathway
FACS: fluorescence-activated cell sorting
GPCR: G protein coupled receptor
ip: intraperitoneally
Lpar1: *lysophosphatidic receptor* *1*
LPL: lysophospholipid
LPLr: lysophospholipid receptor
MPC: multipotent progenitor cell
NICD: Notch Intracellular domain
P1: postnatal day 1
PCA: principal component analysis
PTX: pertussis toxin A
PS: pluripotent stem
qPCR: quantitative polymerase chain reaction
S1p: sphingosine-1-phosphate
S1pr2: S1p receptor 2
Sel1l: Sel-1 suppressor of lin-12-like
Sphk: Sphingosine kinase
YAP: yes-associated-protein
wt: wild type
