# Supplementary figures and images for: Pancreas lineage allocation and specification are regulated by sphingosine-1-phosphate signalling
Source: PLoS Biol. 2017 Mar 1;15(3):e2000949. doi: 10.1371/journal.pbio.2000949 (PMC5331964; doi:10.1371/journal.pbio.2000949)

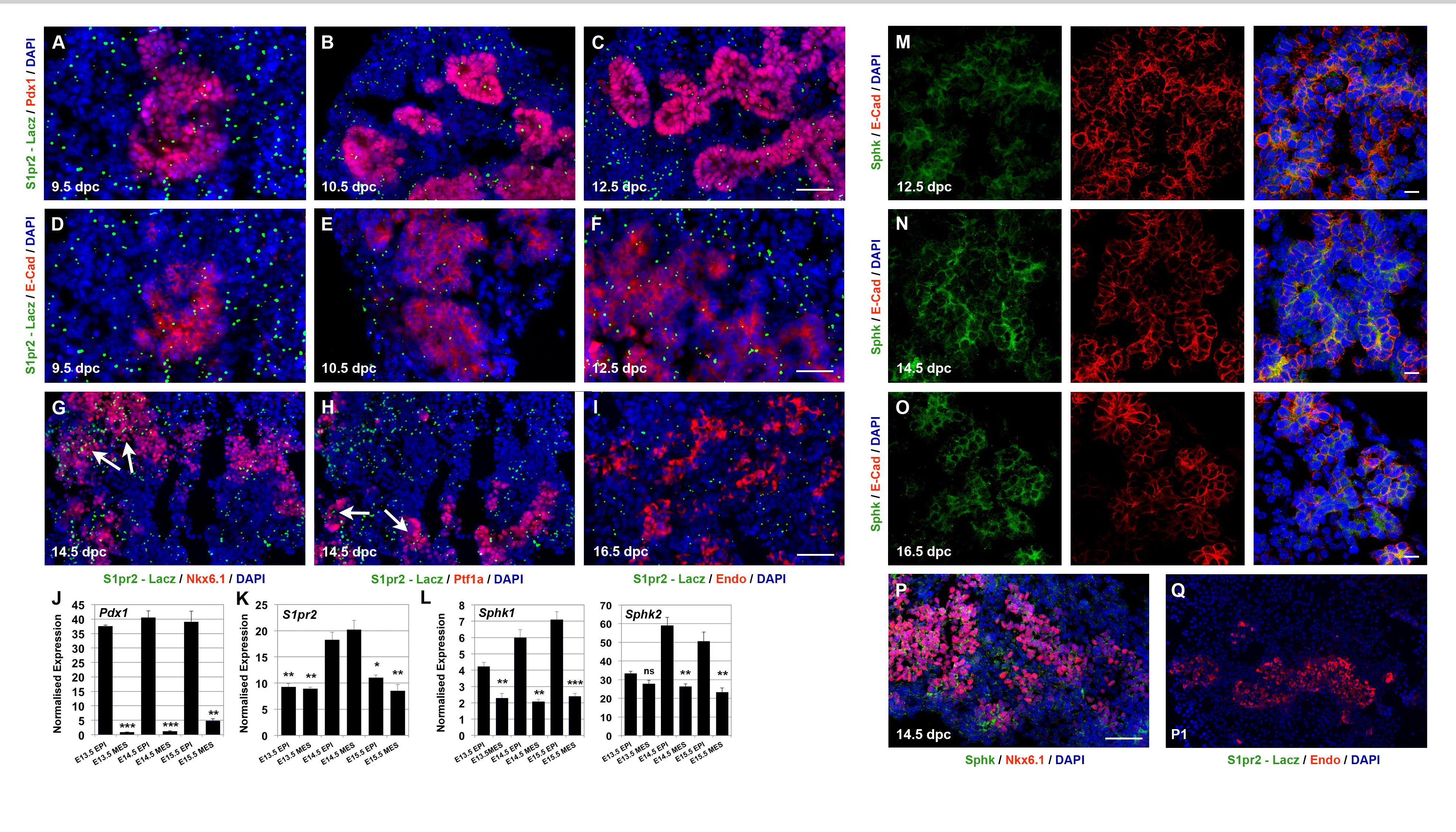

Supplement: S1 Fig — (A-F) Tracking β-galactosidase activity with the X-gal assay from heterozygous S1P2tm1lacz mouse embryos revealed that S1pr2 was expressed in the pancreas at 9.5 dpc (A, D) and was mainly detected in the mesenchyme until 12.5 dpc because immunofluorescence showed that S1pr2 expression was apparent in a few Pdx1+ (B, C) and E-cadherin+ (E, F) epithelial cells. (G-I, Q) Epithelial S1pr2 expression detected by the x-gal activity of the modified allele in heterozygous S1P2tm1lacz mouse embryos peaked at 14.5 dpc, and immunofluorescence showed that it was co-locoalized with Nkx6.1+ trunk cells (eg arrows in G) as well as with Ptf1a+ tip cells (eg arrows in H). The same analysis showed that by 16.5 dpc, S1pr2 expression was eliminated in nascent endocrine C-Pep+ and Gcg+ (Endo) cells and significantly reduced in the rest of the tissue (I). At P1 expression had disappeared completely (Q).(J-L) Quantitative PCR analysis on FACS-isolated epithelial and mesenchymal components of the developing pancreas at 13.5, 14.5 and 15.5 dpc confirmed that S1pr2, Sphk1 and Sphk2 expression peaked at 14.5 dpc (K, L), and that Sphk1 and Sphk2 expression was predominantly epithelial (L). The efficiency of the separation was confirmed independently by determining Pdx1 expression by qPCR in the mesenchymal and epithelial components of FACS separated cells (J). (M-P) Time course of Sphk expression by immunofluorescence at 12.5, 14.5 and 16.5 dpc confirmed the presence of the protein primarily in the epithelium (M-O) and co-localization with Nkx6-1 at 14.5 dpc in the epithelial trunk cells (P). Scale bars, 40μm (A, F), 80μm (G-I, P), 10μm (M-O), 160μm (Q); *p< 0.05, **p<0.01, ***p<0.001, ns not significant in reference to epithelial expression in the corresponding time point (J, L) or corresponding to the E14.5 samples (K); Error bars show SEM. For raw data please refer to the S2 Data file. (TIF) [file pbio.2000949.s001.tif]

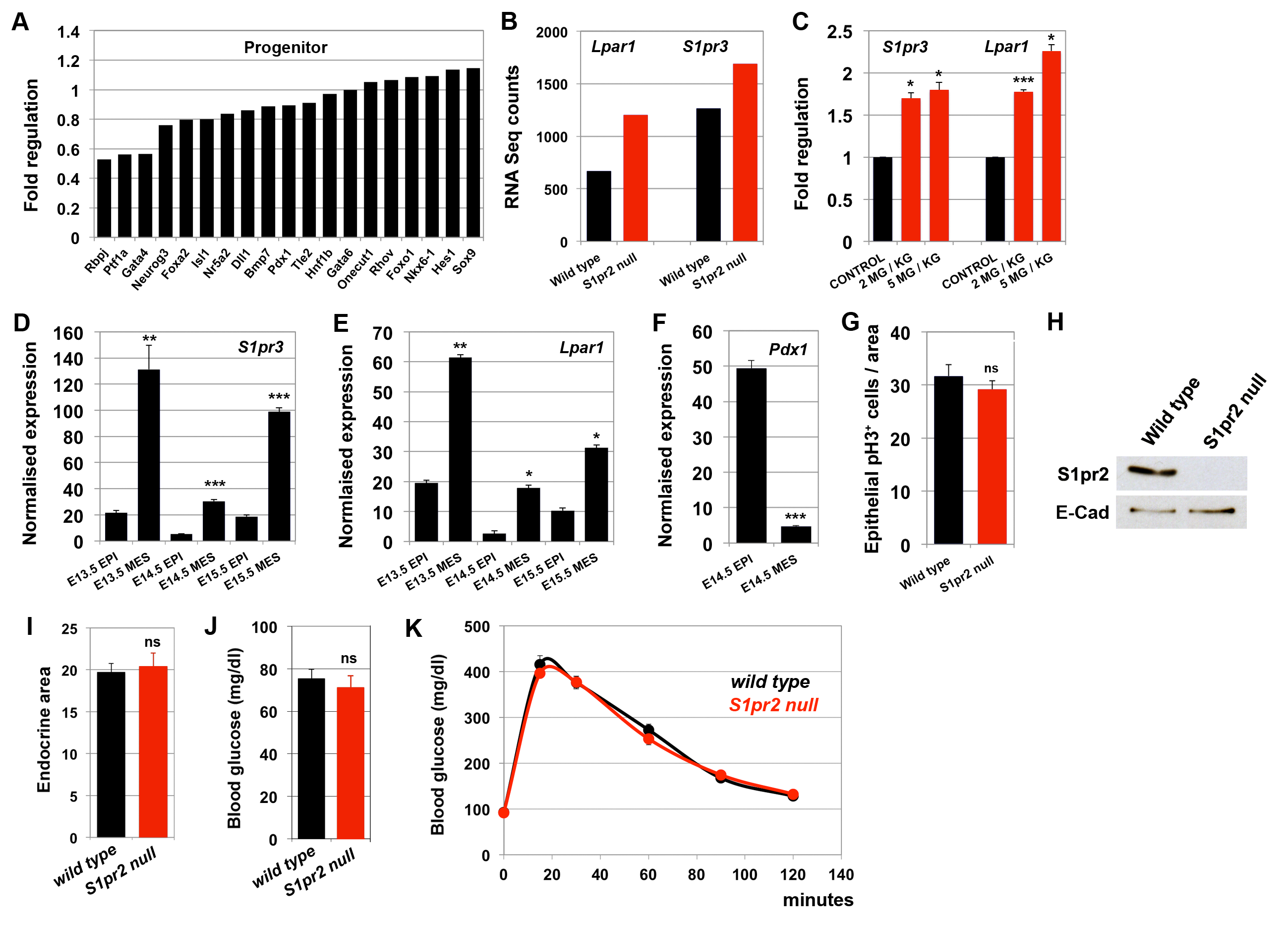

Supplement: S2 Fig — (A-G) RNA Seq gene expression profiling revealed that transcription factors and other genes implicated in epithelial progenitor specification and maintenance were not significantly affected in S1pr2tm1Rlp null pancreata at 14.5 dpc (A). RNA seq counts of both S1pr3 and Lpar1 were increased in the S1pr2tm1Rlpnull pancreata at 14.5 dpc (B). Abrogating S1pr2 signalling by intraperitoneally injecting pregnant mice with 2 or 5 mg/kg of body weight JTE013 at 13.5, 14.5 and 15.5 dpc resulted in upregulation of both S1pr3 and Lpar1 at 16.5 dpc as shown by qPCR (C). Quantitative PCR analysis on isolated epithelial and mesenchymal components of the wt developing pancreas at 13.5, 14.5 and 15.5 dpc showed that expression of expression of both S1pr3 (D) and Lpar1 (E) was predominantly mesenchymal. Quantitative PCR for Pdx1 expression was used to confirm the efficiency of mesenchymal and epithelial separation by FACS (F). Immunofluorescence and quantitation of the ratio pH3+ / E-cadherin+ cells in wt and S1pr2 null pancreata showed that epithelial proliferation was not affected (G). (H) Western blot analysis on wild-type and S1pr2tm1Rlp null pancreata at 14.5 dpc show that S1Pr2 protein is completely absent in the S1pr2tm1Rlpnull. (I-K) The number of endocrine cells (C-pep+ and Gcg+) in S1pr2 null newborns is similar to wt littermates (I) and 8 week S1pr2 null adults show no difference in fasting glucose blood levels (J) or in glucose tolerance test (K). padj < 0.05 (B); *p< 0.05, **p<0.01, ***p<0.001, ns not significant in reference to expression in untreated or control samples; Error bars show SEM. For raw data please refer to the S2 Data file. (TIF) [file pbio.2000949.s002.tif]

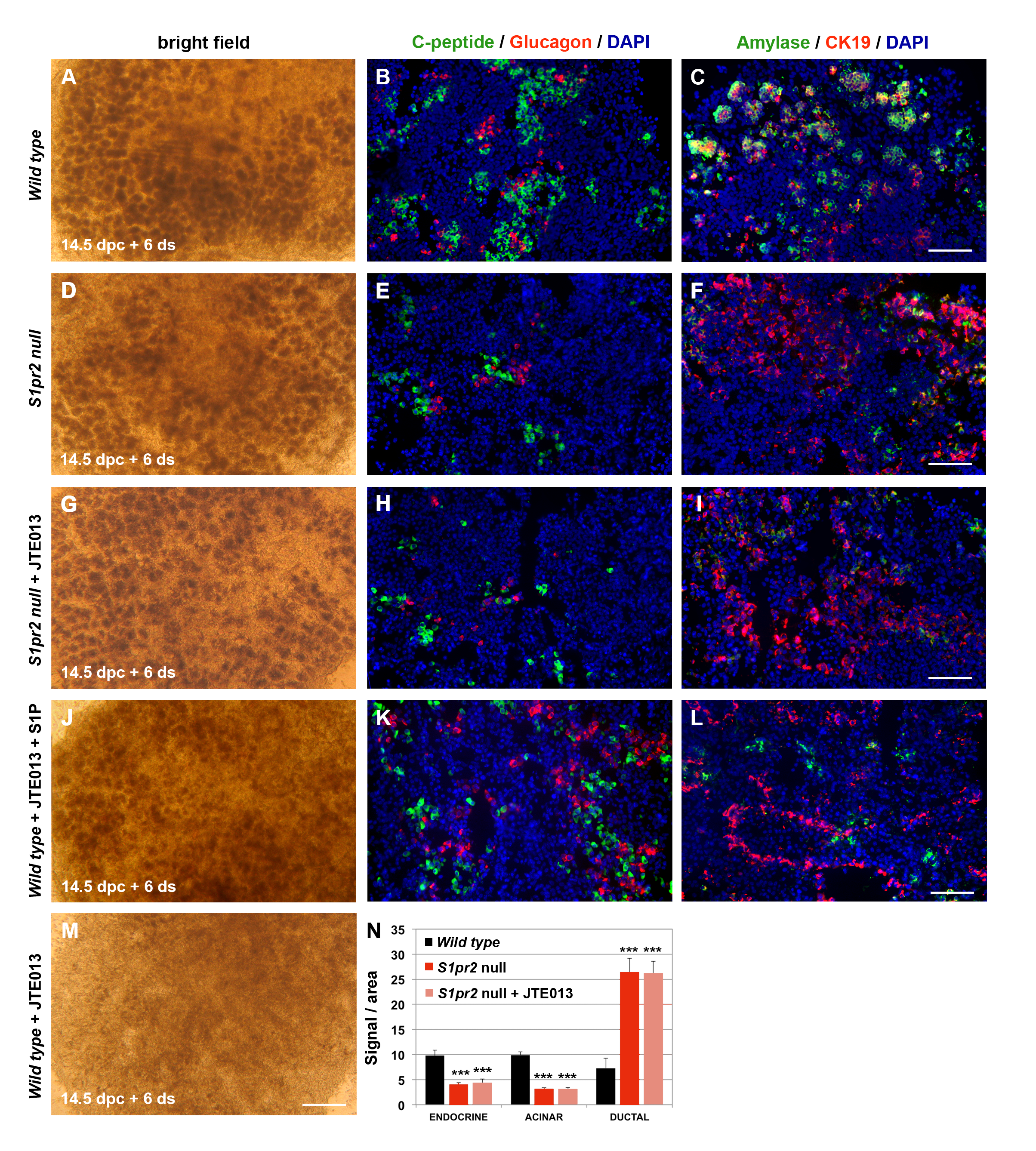

Supplement: S3 Fig — (A-I, M, N) Immunofluorescence analysis showed that 14.5 dpc S1pr2 null pancreata in ALI cultures for 6 days (14.5 dpc + 6ds) gave a strongly reduced number of C-pep+ and Gcg+ endocrine cells (B, E, N), a strongly reduced number of Amy+ acinar cells and an increased number of CK19+ duct-like cells (C, F, N). S1pr2 block by 15μM JTE013 in 14.5 dpc + 6ds ALI cultures of wild-type pancreata resulted in morphological defects characterised by an absence of the dense cell clusters observed by brightfield microscopy in untreated wt and S1pr2 tm1Rlp null cultures (compare M to A and D). In contrast, 14.5 dpc + 6ds ALI cultures of S1pr2 null pancreata in the presence of 15μM JTE013 caused no such morphological defects (G), and immunofluorescence analysis showed that it did not further affect specification of endocrine (C-peptide+ and Glucagon+)(B, E, H, N), acinar (Amylase+) or ductal (CK19+) (C, F, I, N) cells confirming the specificity of JTE013 for S1pr2 also in this context. (J-L) Immunofluorescence analysis showed that the presence of 20 μM S1p rescued specification of endocrine (Cpep+ and Gcg+) cells in JTE013-treated 14.5 dpc + 6 ds ALI cultures (K), and to a lesser extent specification of acinar (Amy+) and ductal (CK19+) cells (L). Morphological defects were also rescued under these conditions as evidenced by brightfield microscopy (J). Quantitations are provided in S7A Fig.Scale bars, 80μm (B, C, E, F, H, I, K, L) and 100μm (A, D, G, J, M); ***p<0.001, in reference to corresponding untreteated controls; Error bars show SD. For raw data please refer to the S2 Data file. (TIF) [file pbio.2000949.s003.tif]

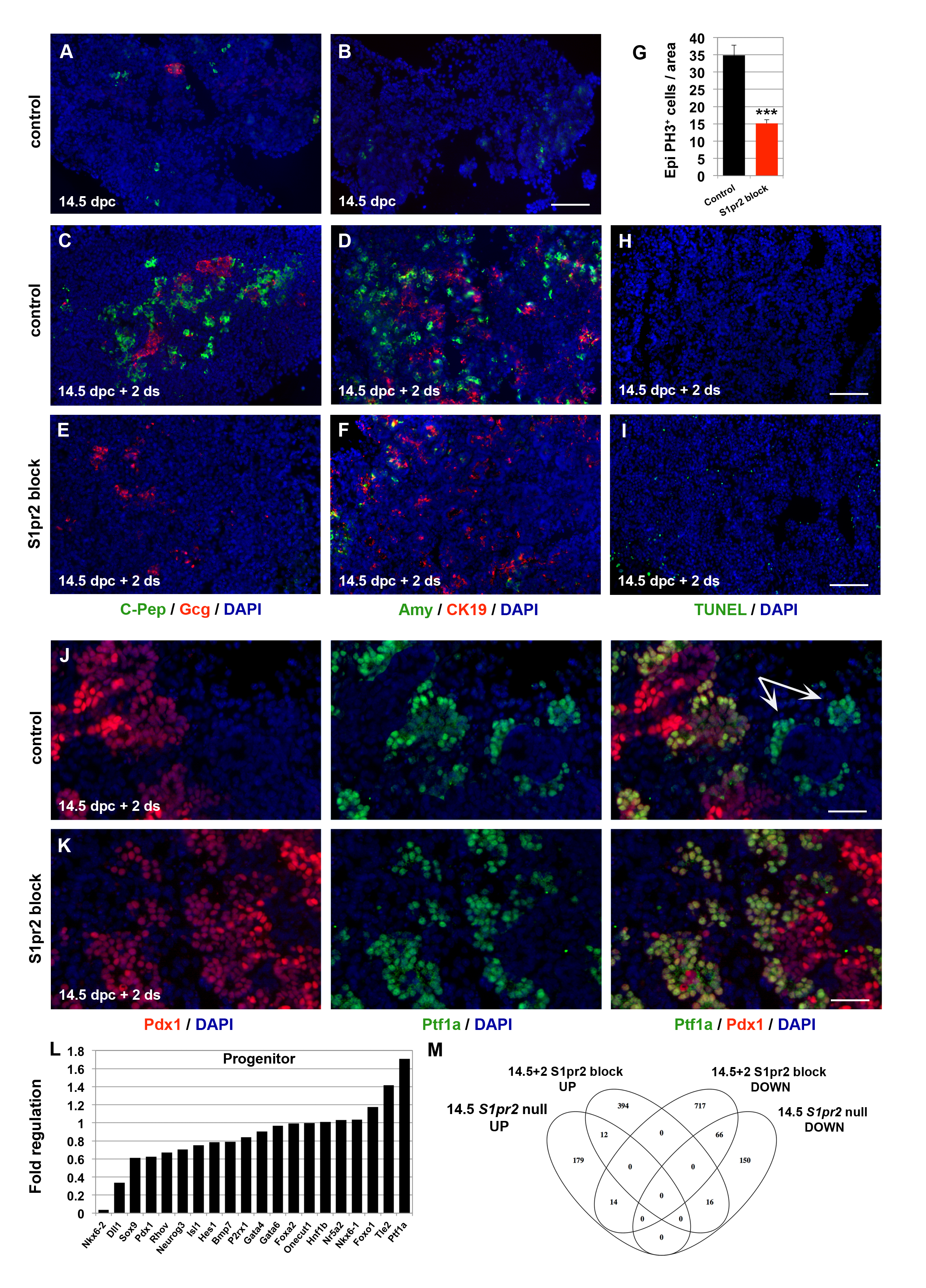

Supplement: S4 Fig — (A-I) Immunofluorescence showed that terminally differentiated endocrine (C-pep+ and Gcg+), acinar (Amy+) and ductal (CK19+) cells are scarce in wt 14.5 dpc embryonic pancreata (A, B), but are readily detected after 2 days in ALI cultures (C, D). S1pr2 block with 15 μM JTE013 in 14.5 dpc + 2 ds ALI cultures and immunofluorescence analysis showed a loss of endocrine (C-pep+) and acinar (Amy+) cells (C-F) and a decrease in epithelial proliferation (ratio of pH3+/E-Cad+ cells) (G). In these experiments a small increase in cell death was evident by TUNEL analysis (H, I). (J, K) High-magnification immunofluorescence showing the presence of Ptf1a+/Pdx1- cells in 14.5 + 2 ds ALI cultures (arrows in J), which are lost upon S1pr2 block (K). (L, M) RNA Seq gene expression analysis on 14.5 dpc + 2 ds pancreatic ALI cultures, revealed that expression of progenitor markers was only weakly affected upon S1pr2 block with 15 μM JTE013, with the notable exception of Nkx6.2 and Dll1 (L). Venn diagram for up- and down-regulated genes in 14.5 dpc S1pr2 tm1Rlp null pancreata and wt pancreata and pancreata cultured for 2 days in standard conditions or with S1pr2 signaling blocked by 15 μM JTE013 (M). Scale bars, 80μm (A-F, I, J), 25μm (J, K). For raw data please refer to the S2 Data file. (TIF) [file pbio.2000949.s004.tif]

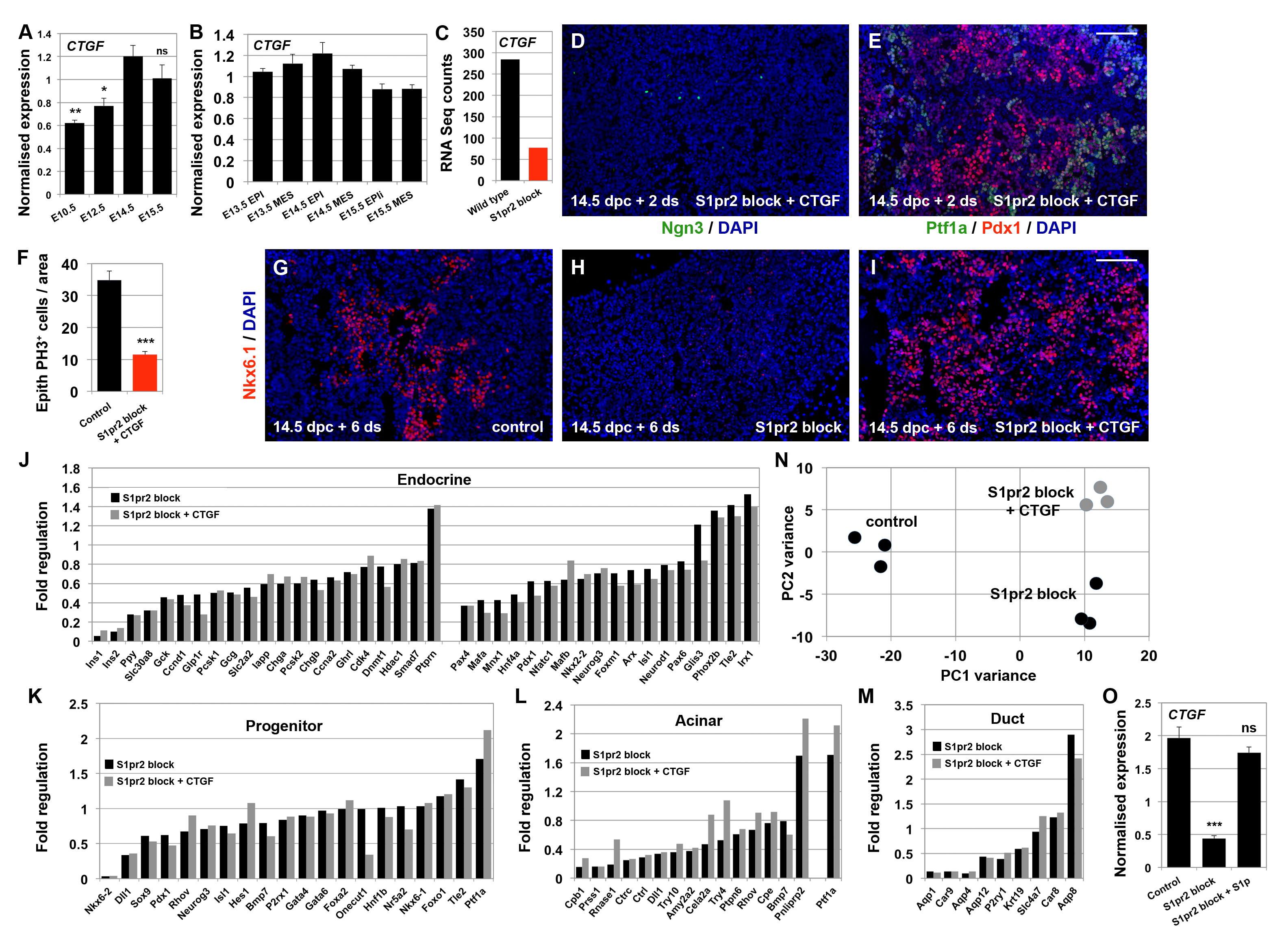

Supplement: S5 Fig — (A-C) Quantitative PCR analysis showed that CTGF expression peaks at 14.5 dpc (A) and that it was expressed in both the mesenchyme and the epithelium of 13.5, 14.5 and 15.5 dpc embryonic pancreata (B). S1pr2 block in 14.5 dpc + 2 ds ALI pancreatic cultures with 15 μM JTE013 causes a 3.5-fold decrease in CTGF expression as shown by RNA Seq (C). (D-I) Immunofluorescence analysis of 14.5 dpc + 2 ds (D-F) or 14.5 dpc + 6 ds (G-I) ALI cultures that were S1pr2 signaling blocked with 15 μM JTE013 and supplemented with 50 ng/ml CTGF. Addition of CTGF was not sufficient to restore the number of Ngn3+ cells (D), there was no effect on the expression pattern of Pdx1 and Ptf1a progenitor markers (E) and epithelial proliferation remained decreased (F). S1pr2 block in 14.5 dpc + 6 ds ALI cultures eliminated Nkx6.1+ cells (G, H) but supplementation with CTGF for the duration of the culture lead to an expanded population of Nkx6.1+ cells (I) (J-N) Comparison of gene expression changes detected by RNA Seq analysis in 14.5 dpc + 2 ds dpc ALI cultures under conditions of S1pr2 block (15 μM JTE013), in the presence (grey bars) or absence (black bars) of CTGF. Gene expression changes (fold regulation) of progenitor (K), endocrine (J), acinar (L) and duct (M) markers were strikingly similar in the two conditions as compared to untreated 14.5 dpc + 2 ds ALI cultures. Principal component analysis (PCA) showed that S1pr2-blocked explants with or without CTGF cluster much closer together than with untreated control explants (N). (O) Quantitative PCR analysis showed that the presence of 20μM S1p in S1pr2-blocked 14.5 dpc + 2 ds ALI cultures restored CTGF expression to control levels. Scale bars, 80μm (D, E, G-I); padj<0.05 (C); *p< 0.05, **p<0.01, ***p<0.001, ns not significant in reference to E14.5 samples (A) or untreated samples (F); Error bars show SEM. For raw data please refer to the S2 Data file. (TIF) [file pbio.2000949.s005.tif]

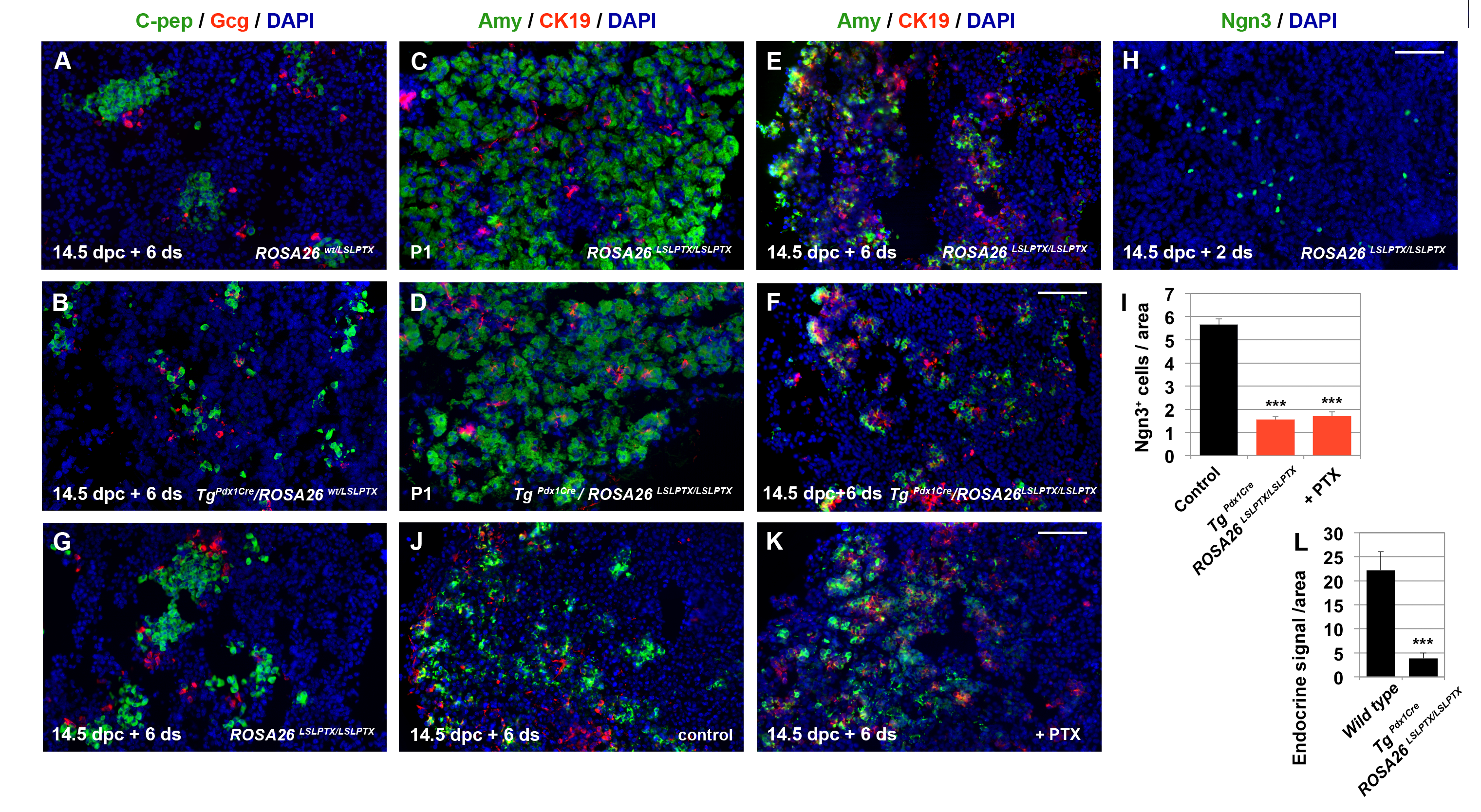

Supplement: S6 Fig — (A-K) Expression of a single allele of the conditional ROSA26LSLPTX transgene in pancreatic epithelial progenitors using the Pdx1-Cre driver TgPdx1Cre resulted in disruption of endocrine cell migration and clustering as shown by immunofluorescence of 14.5 dpc + 6 ds ALI cultures (A, B). Expression of two alleles of the ROSA26LSLPTX transgene using the same driver did not affect Amy+ acinar of CK19+ duct cells either at P1 (C, D) or at 14.5 dpc + 6 ds ALI cultures (E, F, J) as shown by immunofluorescence. Similarly, addition of 10 μg/ml of PTX in 14.5 dpc + 6 ds ALI cultures also had no effect on acinar or duct cell specification (K). Immunofluorescence analysis of 14.5 dpc + 6 ds ALI cultures or 14.5 dpc + 2 ALI cultures from ROSA26LSLPTX/LSL/PTXmice had no defect in endocrine specification and islet clustering (G) or in the generation of Ngn3+ cells (H). Quantitation of the effects of Gαi block in the number of Ngn3+ endocrine progenitor cells showed a strong decrease in both the genetic and ALI culture paradigms (I). (L) Quantitation of the effects of PTX actiivation from the ROSA26 LSLPTX transgene using the TgPdx1Credriver showed a striking loss of C-pep+ and Gcg+ cells compared to wild-type animals at postnatal day 1 (P1). Scale bars, 80μm (A-H, J, K); ***p<0.001, in reference to untreated ALI cultures (I) or wild-type animals (L); Error bars in I show SEM; Error bars in L show SD. For raw data please refer to the S2 Data file. (TIF) [file pbio.2000949.s006.tif]

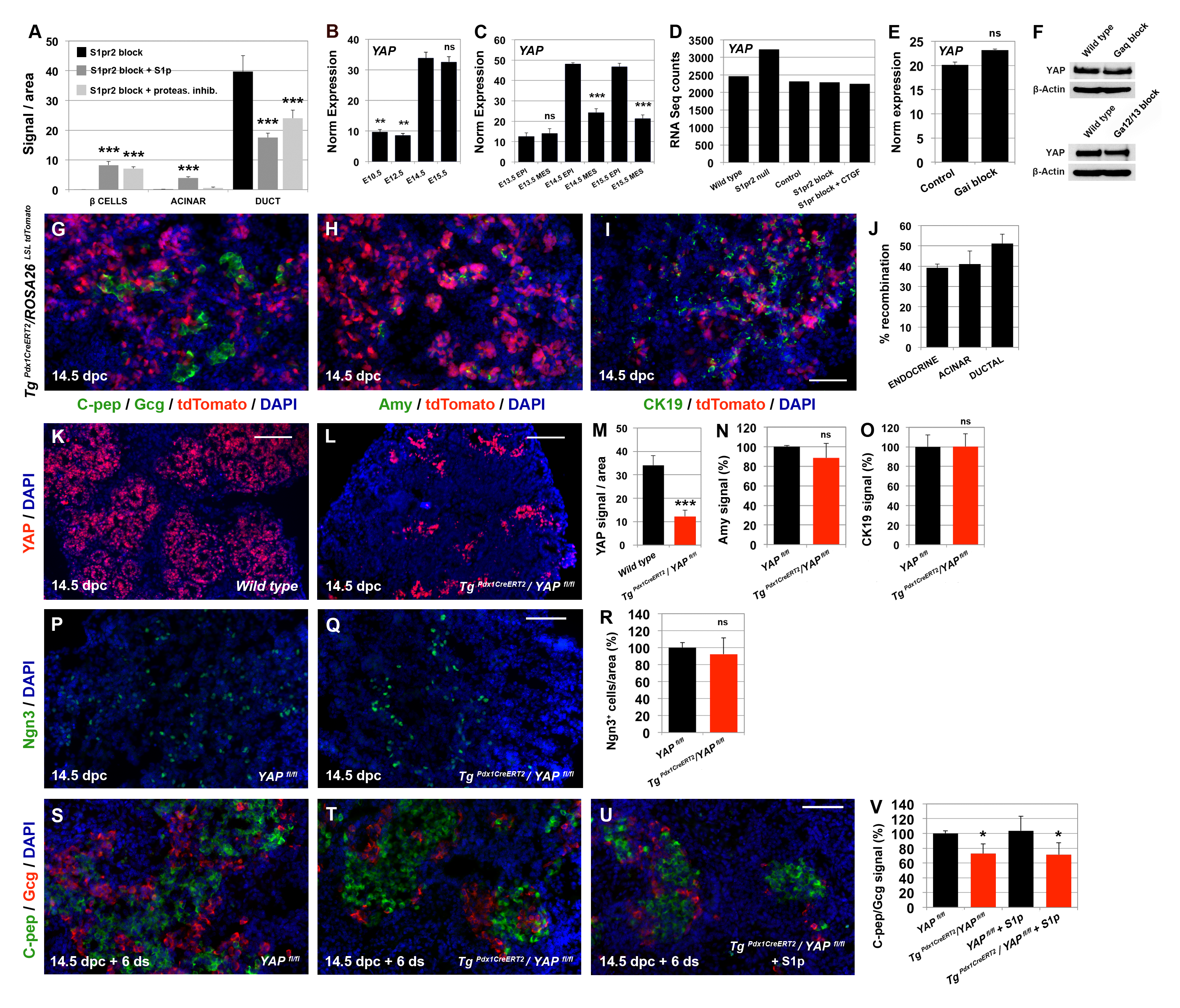

Supplement: S7 Fig — (A) Quantitation of the effects of S1p or the proteasome inhibitor MG132 in S1pr2-blocked 14.5 dpc + 6 ds ALI cultures, on β- (Cpep+), acinar (Amy+) and duct (CK19+) cells. (B) Quantitative PCR analysis in embryonic pancreata at 10.5, 12.5, 14.5 and 15.5 dpc showed that YAP transcript levels peak at 14.5 dpc. (C) Quantitative PCR analysis showed that YAP is expressed in both the mesenchyme and the epithelium of 13.5 embryonic pancreata but is predominantly expressed in the epithelium at 14.5 and 15.5 dpc pancreata. (D) RNA Seq analysis on 14.5 dpc pancreata and pancreatic ALI cultures showed that YAP gene expression is not affected in S1pr2-nulls and in S1pr2-blocked explants in the presence or absence of CTGF (E) Quantitative PCR analysis showed that YAP levels remain unchanged in 14.5 dpc + 2ds ALI cultures treated with 10μg/ml PTX compared to untreated controls. (F) Western blot analysis on 14.5 + 2 ds ALI cultures treated with 50μM of the Gαq inhibitor GP-2A or 5μg/ml of the Gα12/13 inhibitor C3-exoenzyme, showed that YAP levels remained unchanged in both cases, compared to untreated controls.(G-J, N, O) A conditional ROSA26LSLtdTomatoallele was activated using the TgPdx1CreERT2 driver and tamoxifen ip injections in pregnant mice. Analysis by immunofluorescence at 14.5 dpc showed that endocrine (C-pep+ and Gcg+)(G), acinar (Amy+) (H) and ductal (CK19+) (I) cells were labeled with tdTomato, with a similar efficiency ranging between 38–50% (J). Immunofluorescence analysis of these samples showed that there was no effect on acinar (N) or duct (O) cell specification. (K-M) Immunofluorescence analysis on the above samples at 14.5 dpc indicated that YAP expression in TgPdx1CreERT2 / YAPfl/fl pancreata (L, M) drops by 64% compared to their wild-type counterparts (K, M). (P-R) However, no differences in the numbers of Ngn3+ cells were observed between YAP inactivated (Q,R) and control YAPfl/fl pancreata (P,R). (S-V) TgPdx1CreERT2 / YAPfl/fl E14.5 pancreata kept in cultu [file pbio.2000949.s007.tif]

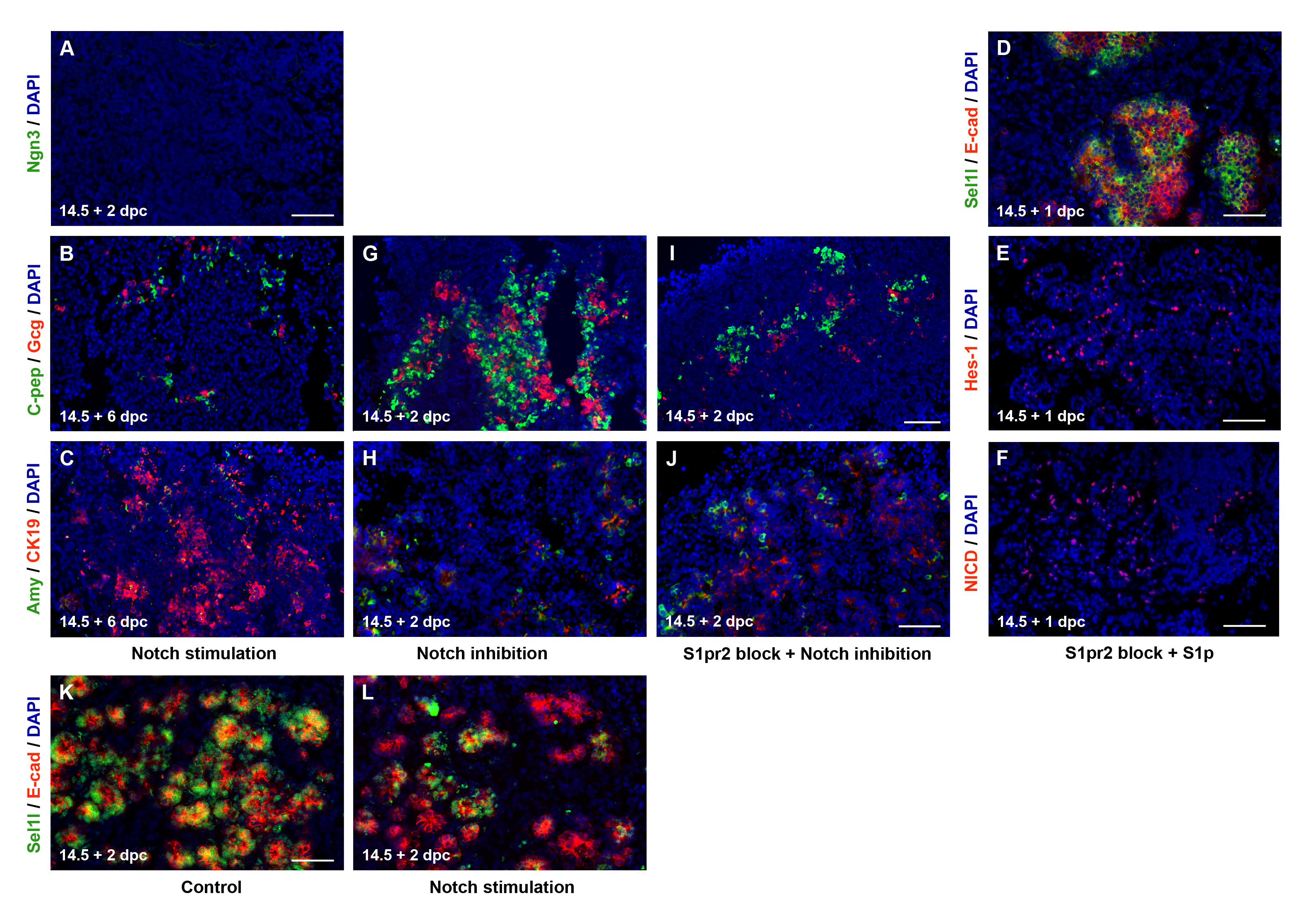

Supplement: S8 Fig — (A-C) Immunofluorecence analysis showed that stimulation of Notch signalling with 10 μM DSL resulted in elimination of Ngn3+ cells (A, quantitations in Fig 8B) in 14.5 dpc + 2ds ALI cultures and in a significant reduction in the number of C-pep+, Gcg+ (B, quantitations in Fig 8C) and Amy+ cells in favour of an expanded population of CK19+ cells (C, quantitations in Fig 8C) in 14.5 dpc + 6ds ALI cultures (D-F) Immunofluorescence analysis in 14.5 dpc + 1d ALI cultures showed that Sel1l expression was restored in the absence of S1pr2 signalling by 20 μM of S1p (D), and this was sufficient to restore attenuation of both Hes1 (E) and NICD (F) expression. (G-H) Notch inhibition of 14.5 dpc + 2 ds ALI cultures with 10 μM DAPT caused a dramatic transient expansion of C-pep+ and Gcg+ cells (G), at the expense of Amy+ and CK19+ cells (H). (I-J) Notch inhibition with 10 μM DAPT together with S1pr2 block with 15 μM JTE013 in 14.5 dpc + 2 ds ALI cultures transiently restored endocrine (C-pep+) (I) and acinar (Amy+) (J) cell specification. (K-L) Immunofluorescence analysis in 14.5 dpc + 2d ALI cultures showed that Notch stimulation with 10 μM DSL resulted in a significant reduction of Sel1l expression (K, L). Scale bars, 70μm (A-C and G-J), 50μm (D-F, K, L). For raw data please refer to the S2 Data file. (TIF) [file pbio.2000949.s008.tif]
