# Supplementary material for: Pancreas lineage allocation and specification are regulated by sphingosine-1-phosphate signalling
Source: PLoS Biol. 2017 Mar 1;15(3):e2000949. doi: 10.1371/journal.pbio.2000949 (PMC5331964; doi:10.1371/journal.pbio.2000949)
Supplement: S3 Table — (DOC) [file pbio.2000949.s011.doc]

| **Official Nomenclature** | **Abbreviation** | **Description** | **Ref** |
| --- | --- | --- | --- |
| *S1P2^tm2Ytak^* | *S1pr2^tm1lacZ^* | Functional inactivation with lacZ insertion | 1 |
| *YAP^fl/fl^* | *YAP^fl/fl^* | Conditional deletion of exons1 and 2 | 2 |
| *Tg^Pdx1CreERT2^* | *Tg^Pdx1CreERT2^* | Transgene expressing CreERT2 under Pdx1 (Pancreatic and Duodenal Homeobox 1) promoter | 3 |
| *S1pr2^tm1Rlp^* | *S1pr2* null | Disruption of exon1 through insertion of a Neo cassette | 4 |
| *Gt(ROSA)26Sor^tm1(ptxA)Cgh^* | *ROSA26^LSLPTX^* | Knock-in of ptxA (pertussis toxin subunit 1) for expression after Cre-mediated recombination | 5 |
| *Gt(ROSA)26Sor^tm9(CAG-tdTomato)hZE^* | *ROSA26^LSLTdTomato^* | Reporter line which expresses TdTomato following Cre-mediated recombination | 6 |
| *Tg^(Pdx1-cre)6Tuv^* | *Tg^Pdx1Cre^* | Transgene expressing Cre under Pdx1 promoter | 7 |

**Table S3** List of mouse lines used
