# Supplementary material for: Pancreas lineage allocation and specification are regulated by sphingosine-1-phosphate signalling
Source: PLoS Biol. 2017 Mar 1;15(3):e2000949. doi: 10.1371/journal.pbio.2000949 (PMC5331964; doi:10.1371/journal.pbio.2000949)
Supplement: S4 Table — (DOC) [file pbio.2000949.s012.doc]

**Table S4. Genotyping primers and PCR Conditions**

**Reaction for *Gt(ROSA)26Sor^tm1(ptxA)Cgh^***

Fwd (Common): AAAGTCGCTCTGAGTTGTTAT

Rev1 (WT): GGAGCGGGAGAAATGGATATG

Rev2 (Transgenic): GCGAAGAGTTTGTCCTCAACC

| Step | Temp | Time | Notes |
| --- | --- | --- | --- |
| 1 | 95ºC | 5 min |  |
| 2 | 95ºC | 30 s |  |
| 3 | 58ºC | 30 s |  |
| 4 | 72ºC | 60 s | Repeat steps 2-4 for 40 cycles |
| 5 | 65ºC | 10 min |  |
| 6 | END |  |  |

Product size WT: 550 bp, Transgenic: 350 bp

**Reaction for *Gt(ROSA)26Sor^tm9(CAG-tdTomato)hZE^***

Fwd1: AAGGGAGCTGCAGTGGAGTA

Rev1: CCGAAAATCTGTGGGAAGTC

Fwd2: GGCATTAAAGCAGCGTATCC

Rev2: CTGTTCCTGTACGGCATGG

| Step | Temp | Time | Notes |
| --- | --- | --- | --- |
| 1 | 94ºC | 2 min |  |
| 2 | 92ºC | 40 s |  |
| 3 | 58ºC | 40 s |  |
| 4 | 72ºC | 45 s | Repeat steps 2-4 for 40 cycles |
| 5 | 72ºC | 5 min |  |
| 6 | END |  |  |

Product size WT: 297 bp, Transgenic: 196 bp

**Reaction for *Tg^(Pdx1-cre)6Tuv^* and *Tg ^Pdx1CreERT2^* for Cre**

Fwd: CTGCCACGACCAAGTGACAGC

Rev: GCTAAGTGCCTTCTCTACACCTGC

| Step | Temp | Time | Notes |
| --- | --- | --- | --- |
| 1 | 94 ºC | 3 min |  |
| 2 | 94ºC | 30 s |  |
| 3 | 61ºC | 30 s |  |
| 4 | 72ºC | 45 s | Repeat steps 2-4 for 35 cycles |
| 5 | 72ºC | 7 min |  |
| 6 | END |  |  |

Product size Cre: 333 bp

**Reaction for *YAP^fl/fl^***

Fwd: CCATTTGTCCTCATCTCTTACTAAC

Rev: GATTGGGCACTGTCAATTAATGGGCTT

| Step | Temp | Time | Notes |
| --- | --- | --- | --- |
| 1 | 94ºC | 1 min |  |
| 2 | 59ºC | 1min |  |
| 3 | 72ºC | 1min | Repeat steps 1-3 for 2 cycles |
| 4 | 92ºC | 1min |  |
| 5 | 59ºC | 1min |  |
| 6 | 72ºC | 1min | Repeat steps 4-6 for 35 cycles |
| 7 | 72ºC | 10min |  |
| 8 | END |  |  |

Primer size WT: 498 bp Transgenic: 597 bp

**Reaction for *S1pr2^tm1Rlp^***

Fwd (Common): GCAGTGACAAAAGCTGCCGAATGCTGATG

Rev1 (WT): AGATGGTGACCACGCAGAGCACGTAGTG

Rev2 (Mutant): TGACCGCTTCCTCGTGCTTTACGGTATCG

| Step | Temp | Time | Notes |
| --- | --- | --- | --- |
| 1 | 95 ºC | 5 min |  |
| 2 | 95ºC | 30 s |  |
| 3 | 55ºC | 60 s |  |
| 4 | 72ºC | 60 s | Repeat steps 2-4 for 35 cycles |
| 5 | 72ºC | 7 min |  |
| 6 | END |  |  |

Primer size WT: 170 bp, Mutant: 220 bp

**Reaction for *S1P2^tm2Ytak^***

Fwd1 (WT): cagtgacaaaagctgccgaatgctgatgct

Fwd2 (Mutant): tggctacccgtgatattgctgaagagcttg

Rev (Common): tgagcagtgagttaagggtggcaaaggcaa

| Step | Temp | Time | Notes |
| --- | --- | --- | --- |
| 1 | 94 ºC | 5 min |  |
| 2 | 94ºC | 30 s |  |
| 3 | 59ºC | 45 s |  |
| 4 | 72ºC | 30 s | Repeat steps 2-4 for 33 cycles |
| 5 | 72ºC | 7 min |  |
| 6 | END |  |  |

Primer size WT: 424 bp, Mutant: 241 bp
